# Impact of *Bmal1* Rescue and Time-Restricted Feeding on Liver and Muscle Proteomes During the Active Phase in Mice

**DOI:** 10.1016/j.mcpro.2023.100655

**Published:** 2023-10-02

**Authors:** Jacob G. Smith, Jeffrey Molendijk, Ronnie Blazev, Wan Hsi Chen, Qing Zhang, Christopher Litwin, Valentina M. Zinna, Patrick-Simon Welz, Salvador Aznar Benitah, Carolina M. Greco, Paolo Sassone-Corsi, Pura Muñoz-Cánoves, Benjamin L. Parker, Kevin B. Koronowski

**Affiliations:** 1 Department of Medical and Life Sciences (MELIS), Pompeu Fabra University (UPF), Parc de Recerca Biomèdica de Barcelona (PRBB), Barcelona, Spain; 2 Department of Anatomy and Physiology, Centre for Muscle Research, The University of Melbourne, Melbourne, Victoria, Australia; 3 Department of Radiation Oncology, Mays Cancer Center at UT Health San Antonio MD Anderson, Joe R. and Teresa Lozano Long School of Medicine, San Antonio, Texas, USA; 4 Barshop Institute for Longevity and Aging Studies at UT Health San Antonio, San Antonio, Texas, USA; 5 Department of Biochemistry & Structural Biology, University of Texas Health San Antonio, San Antonio, Texas, USA; 6 Institute for Research in Biomedicine (IRB Barcelona), The Barcelona Institute of Science and Technology (BIST), Barcelona, Spain; 7 Hospital del Mar Research Institute Barcelona, Cancer Research Program, Barcelona Biomedical Research Park (PRBB), Barcelona, Spain; 8 Catalan Institution for Research and Advanced Studies (ICREA), Barcelona, Spain; 9 Department of Biomedical Sciences, Humanitas University, Milan, Italy; 10 IRCCS Humanitas Research Hospital, Rozzano, Milan, Italy; 11 Department of Biological Chemistry, Center for Epigenetics and Metabolism, U1233 INSERM, University of California, Irvine, California, USA; 12 Altos Labs, Inc, San Diego Institute of Science, San Diego, California, USA

**Keywords:** circadian clocks, circadian rhythms, *Bmal1*, liver, muscle, time-restricted feeding, fibroblast growth factor, FGF1

## Abstract

Molecular clocks and daily feeding cycles support metabolism in peripheral tissues. Although the roles of local clocks and feeding are well defined at the transcriptional level, their impact on governing protein abundance in peripheral tissues is unclear. Here, we determine the relative contributions of local molecular clocks and daily feeding cycles on liver and muscle proteomes during the active phase in mice. LC–MS/MS was performed on liver and gastrocnemius muscle harvested 4 h into the dark phase from WT, Bmal1 KO, and dual liver- and muscle-Bmal1–rescued mice under either ad libitum feeding or time-restricted feeding during the dark phase. Feeding-fasting cycles had only minimal effects on levels of liver proteins and few, if any, on the muscle proteome. In contrast, Bmal1 KO altered the abundance of 674 proteins in liver and 80 proteins in muscle. Local rescue of liver and muscle Bmal1 restored ∼50% of proteins in liver and ∼25% in muscle. These included proteins involved in fatty acid oxidation in liver and carbohydrate metabolism in muscle. For liver, proteins involved in de novo lipogenesis were largely dependent on Bmal1 function in other tissues (i.e., the wider clock system). Proteins regulated by BMAL1 in liver and muscle were enriched for secreted proteins. We found that the abundance of fibroblast growth factor 1, a liver secreted protein, requires BMAL1 and that autocrine fibroblast growth factor 1 signaling modulates mitochondrial respiration in hepatocytes. In liver and muscle, BMAL1 is a more potent regulator of dark phase proteomes than daily feeding cycles, highlighting the need to assess protein levels in addition to mRNA when investigating clock mechanisms. The proteome is more extensively regulated by BMAL1 in liver than in muscle, and many metabolic pathways in peripheral tissues are reliant on the function of the clock system as a whole.

## Highlights

• The clock gene *Bmal1* regulates more proteins than time-restricted feeding at ZT16.
• Liver *de novo* lipogenesis machinery requires clock function in other tissues.
• Secreted proteins are enriched among *Bmal1*-regulated proteins.
• Fibroblast growth factor 1 modulates mitochondrial respiration in hepatocytes *via* autocrine signaling.

## Graphical Abstract

**Figure:**
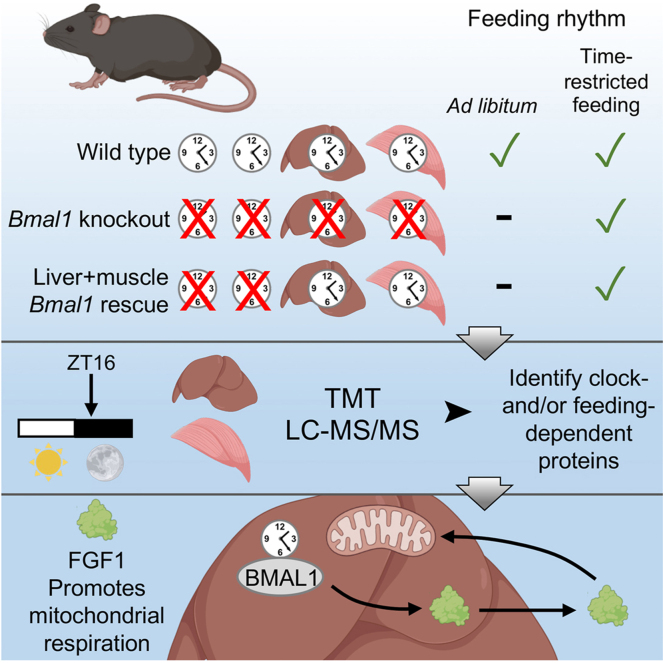

## In Brief

Circadian clock genes and feeding cycles regulate the transcriptome of metabolic tissues. Here, we used tandem mass tag labeling combined with LC–MS/MS to interrogate regulation of the proteome and show that, at nighttime, the effect of knocking out the molecular clock component *Bmal1* is substantially greater than the effect of time-restricted feeding. In addition, we show that *Bmal1*-dependent proteins are enriched for secreted proteins, such as fibroblast growth factor 1, which modulates mitochondrial respiration in hepatocytes.

The mammalian circadian clock system drives 24 h rhythms of physiology, coordinating metabolic processes within and between tissues. Disruption of the clock system *via* genetic or environmental means induces metabolic dysregulation and increases risk for metabolic disease (1, 2, 3). The central clock in the suprachiasmatic nucleus regulates systems-level coordination through behavioral cycles, including locomotion and feeding-fasting behavior, which cooperate with humoral and neuronal mechanisms to signal to peripheral tissue clocks. While daily transcriptional programs that support metabolic function are dependent on this coordination, it remains unclear how local and central clock signals interact at the proteomic level.

Two main components of circadian control in peripheral tissues have been described. The local autonomous clock controls a subset of transcriptional rhythms in each tissue, with metabolic function in each tissue similarly restricted (4, 5, 6), and the centrally driven feeding-fasting rhythm engages systemic hormonal signals (*e.g.*, insulin release from the pancreas (5, 7, 8)) and regulates transcription through nutrient responsive signaling pathways (5, 9). Modulation of feeding-fasting cycles through time-restricted feeding (TRF) has numerous beneficial effects on metabolism, such as improved insulin sensitivity and reduction of adiposity. Mouse studies show that TRF has both clock-dependent and clock-independent effects (10, 11). Indeed, our recent work has shown that local clocks and feeding cycles interact to bolster rhythms in liver and muscle and that peripheral clock function is shaped by interactions with other peripheral clocks (12). For example, the glucose intolerance of *Bmal1*-null mice is most improved when both liver and muscle clocks are rescued and engaged with feeding cycles. Thus, various levels of clock control and their interactions are important for metabolic homeostasis.

Most studies, including our own, have focused on transcription using mRNA analyses. However, there are many regulatory steps between transcriptional output and protein products, and post-transcriptional mechanisms of clock control are evident (13, 14, 15). Few studies using clock mutant mice, especially *Bmal1*-null mice, have interrogated clock mechanisms at the protein level (16, 17, 18); accordingly, much less is known about how the clock system controls the proteomic landscape in each tissue. Here, we address how local clocks and feeding cycles impact metabolic pathways at the protein level in liver and muscle. By considering this critical juncture, we move closer to understanding how TRF and clock function may be targeted for therapeutic gain.

## Experimental Procedures

### Animal Experiments

Mice were bred and housed in a vivarium at the University of California Irvine. All animal experiments complied with Animal Research: Reporting of *In Vivo* Experiments (ARRIVE) guidelines and were carried out in accordance with the National Research Council’s Guide for the Care and Use of Laboratory Animals. The study was conducted with approval from the local Institutional Animal Care and Use Committee. *Bmal1*-stop-full length (FL) mice were generated on the C57BL/6J background as previously described (4, 5, 19). Crosses of *Bmal1*-stop-FL mice with Alfp-Cre and Hsa-Cre lines generated mice with reconstitution of *Bmal1* in hepatocytes and cells that constitute skeletal muscle myofibers, respectively (liver and muscle reconstituted [LMRE]). Experimental genotypes were (1) WT—*Bmal1*^*wt/wt*^, *Alfp-*cre^tg/0^, and *Hsa-*cre^tg/0^; (2) *Bmal1* whole-body KO—*Bmal1*^*stop-FL/stop-FL*^, *Alfp-*cre^0/0^, and *Hsa-*cre^0/0^; and (3) *Bmal1* LMRE—*Bmal1*^*stop-FL/stop-FL*^, *Alfp*-cre^tg/0^, and *Hsa*-cre^tg/0^. Additional experiments featured samples from WT—*Bmal1*^*wt/wt*^, *Alfp-*cre^tg/0^, *Bmal1* whole-body KO—*Bmal1*^*stop-FL/stop-FL*^, *Bmal1* liver-reconstituted mice (LRE)—*Bmal1*^*stop-FL/stop-FL*^; *Alfp*-cre^tg/0^ harvested at Barcelona Science Park, Spain, in accordance with the European Union and Spanish regulations. Animal care and experimental use were approved by the government of Catalonia, Spain, in line with national and local legislation. All experiments utilized male mice, aged 8 to 14 weeks old, entrained to a 12 h light:12 h dark cycle. Mice were fed a standard chow diet with access either ad libitum (AL) or in a TRF condition in which mice had food access only during the 12 h dark period from zeitgeber time (ZT) 12 to 24/0 (TRF). Following euthanasia by carbon dioxide, performed in line with local and national guidelines, tissue samples were excised and immediately snap frozen in liquid nitrogen. Experimental conditions were standardised at both institutions, and all mice were derived from the same founder line.

### Proteomic Sample Preparation

Preparation of liver and muscle for proteomic analysis was performed essentially as described previously (20, 21). Briefly, tissue was lysed in 6 M guanidine in 100 mM Tris, pH 8.5 containing 10 mM Tris(2-carboxyethyl)phosphine and 40 mM 2-chloroacetamide but tip-probe sonication and heated at 95 °C for 5 min. The samples were centrifuged at 20,000*g* for 30 min at 4 °C, and the supernatant containing protein was diluted with 1 volume of water and then precipitated with 4 volumes of acetone overnight at −30 °C. Protein was pelleted by centrifugation at 4000*g* for 5 min at 4 °C and washed with 80% acetone. The protein pellet was dried briefly to remove residual acetone and resuspended in 10% trifluoroethanol in 100 mM Hepes (pH 7.5). Proteins were quantified with bicinchoninic acid, normalized to 10 μg/10 μl, and digested overnight with 0.2 μg of sequencing-grade trypsin (Sigma) and 0.2 μg of sequencing-grade LysC (Wako) overnight at 37 °C. Separate pooled samples of liver and muscle were also generated by mixing 4 μg of each sample and aliquoted into 10 μg/10 μl prior to digestion for use as internal controls. The peptides were labeled directly with 20 μg of 10-plex tandem mass tag (TMT) (Thermo Scientific) for 1.5 h at room temperature. A total of six TMT labeling experiments (three for liver and three for muscle) were performed. The labeling scheme and experimental design is uploaded to the Pride Proteomics Repository (see the Data Availability section). After labeling, the reaction was deacylated with a final concentration of 0.2% hydroxylamine for 15 min followed by acidification to a final concentration of 1% TFA, and the samples for each TMT experiment were pooled. Samples were desalted directly with SDB-RPS microcolumns as previously described (21) and dried by vacuum centrifugation. Peptides were resuspended in 2% acetonitrile (methyl cyanide [MeCN]) containing 0.1% TFA and fractionated by neutral pH reversed-phase chromatography on a Dionex 3500 micro-UHPLC. Peptides were injected onto a 0.3 mm × 15 cm column (C18BEH; 1.7 μm; Waters) maintained at room temperature and separated over 60 min gradient of 2 to 40% buffer B at 6 μl/min into 48 fractions and automatically concatenated into 12 fractions in a looping fashion (buffer A: 10 mM ammonium formate [pH 7.5]; buffer B: 90% MeCN). Peptides were dried by vacuum centrifugation and stored at −80 °C.

### Proteomic Analysis and Data Processing

Peptides were resuspended in 2% MeCN containing 0.1% TFA and separated on a nano-Dionex 3500 UHPLC. Peptides were injected onto a 0.075 mm × 40 cm column (C18AQ; 1.9 μm; Dr Maisch, packed into PepSep) maintained at 40 °C and separated over 60 min gradient of 2 to 30% buffer B at 300 nl/min. Peptides were detected on an Orbitrap Eclipse mass spectrometer (ThermoFisher Scientific) *via* electrospray ionization in positive mode with 1.9 kV at 275 °C and RF set to 30%. The instrument was operated in data-dependent acquisition mode with an MS1 spectrum acquired over the mass range 350 to 1550 *m/z* (120,000 resolution, 8 × 10^5^ automatic gain control, and 50 ms maximum injection time) followed by MS–MS analysis with fixed cycle time of 3 s *via* high-energy collisional dissociation fragmentation mode and detection in the Orbitrap (50,000 resolution; 1 × 10^5^ automatic gain control, 86 ms maximum injection time, and 0.7 *m/z* isolation width). Only ions with charge state 2 to 7 triggered MS–MS with peptide monoisotopic precursor selection and dynamic exclusion enabled for 30 s at 10 ppm. Data-dependent acquisition data were searched against the UniProt mouse database (October 2020; UP000000589_10090 and UP000000589_10090_additional with a total of 63,737 entries) with MaxQuant, version 1.6.17.0, using default parameters with peptide spectral matches, peptide and protein false discovery rate set to 1% (22). All data were searched with oxidation of methionine set as the variable modification and carbamidomethylation of cysteine and 10-plex TMT of peptide N termini and lysine set as fixed modifications. First search MS1 mass tolerance was set to 20 ppm followed by recalibration, and main search MS1 tolerance was set to 4.5 ppm, whereas MS–MS mass tolerance was set to 20 ppm. MaxQuant output data were initially processed with Perseus (23) to remove decoy data, potential contaminants, and proteins only identified with a single peptide containing oxidized methionine.

### Cell Culture

Alpha mouse liver 12 (AML12) cells were purchased from the American Type Culture Collection (CRL-2254). Cells were cultured in Dulbecco’s Modified Eagle’s Medium: F12 (American Type Culture Collection, catalog no.:30-2006) supplemented with 10% fetal bovine serum (Corning; catalog no.: 35-015-CV), 1% penicillin–streptomycin, 1× insulin–transferrin–selenium supplement (Corning; catalog no.: 25-800-CR), and 40 ng/ml dexamethasone. Cells were plated at confluency (40,000 cells per well) in Seahorse XF96 Cell Culture Microplates (Agilent). Cells were transfected with siRNA or mammalian expression vectors at the time of plating (reverse transfection). Cells were assayed 48 h later. AZD4547 (Abcam; catalog no.: ab216311) was administered 24 h prior to downstream measurements. The vehicle control was 0.013% dimethyl sulfoxide.

### Oxygen Consumption and Extracellular Acidification Rates

Metabolic rates were measured using the Seahorse XFe96 Analyzer (Agilent) and Seahorse XF Cell Energy Phenotype Test Kit (Agilent; catalog no.: 103325-100) according to the manufacturer’s instructions.

### Fibroblast Growth Factor 1 ELISA Assay

Serum fibroblast growth factor 1 (FGF1) protein was measured with the mouse aFGF ELISA kit (RayBiotech; catalog no.: ELM-aFGF-1) according to the manufacturer’s instructions.

### Western Blot

Livers were homogenized in radioimmunoprecipitation assay buffer (Thermo Scientific; catalog no.: J62524.AE) supplemented with protease inhibitors, lysed on ice for 10 min, sonicated (5 s on, 5 s off, for four cycles at 40% amplitude), centrifuged at maximum speed at 4 °C for 10 min, and then the supernatant was collected. About 20 μg protein (or amount as otherwise indicated) was separated on a 4 to 20% gradient gel by SDS-PAGE and then transferred to a polyvinylidene difluoride membrane. Blots were blocked for 1 h with 5% milk in Tris-buffered saline with 0.1% Tween-20 (TBST) at room temperature. Primary antibodies were diluted in 5% milk TBST and incubated with blots overnight at 4 °C (FGF1—Abcam, catalog no.: ab207321; β-actin—Abcam, catalog no.: ab8226; and albumin—Bethyl Laboratories, catalog no.: A90-134A). Blots were washed three times with TBST and then incubated with horseradish peroxidase–conjugated secondary antibodies (MilliporeSigma, catalog nos.: AP160P and 12-348) for 1 h at room temperature. Blots were then washed three times, incubated with horseradish peroxidase substrate (Millipore; catalog no.: WBLUC0500) for 5 min at room temperature, and visualized using a chemiluminescent imaging system (Bio-Rad). Blots were quantified using ImageJ software (24). Where indicated, total protein on gels was visualized using stain-free gel technologies (Bio-Rad). For *ex vivo* secreted protein experiments, the concentrated supernatant entered the aforementined protocol after the maximum speed centrifugation step.

### Real-Time Quantitative PCR

RNA was extracted using the RNeasy Plus Mini Kit (Qiagen; catalog no.: 74136), and 500 ng of RNA was reverse transcribed using the Maxima First Strand cDNA Synthesis Kit for RT–quantitative PCR (qPCR) (Thermo Scientific; catalog no.: K1641). qPCR was performed using PowerUP SYBR Green Master Mix (Applied Biosystems; catalog no.: A25742) on a QuantStudio 5 (Applied Biosystems). Gene expression data were normalized to 18S ribosomal RNA. Primer sequences were as follows: mouse *Fgf1* forward 5′-ACACCGAAGGGCTTTTATACG-3′ and reverse 5′-GTGTAAGTGTTATAATGGTTTTCTTCCA-3’.

### Secretion of FGF1 from Liver *Ex Vivo*

Secretion of proteins from liver was achieved similarly to previously published methods (25). Mice were anesthetized with vaporized isoflurane (1–4%) and perfused (transcardiac) with 5 ml of PBS to remove blood from within the liver. The median lobe of the liver was harvested, washed in PBS, and transferred to a conical tube containing 5 ml of 1× CD Chinese hamster ovary medium (Gibco; catalog no.: 10743011) bubbled with carbogen. Liver was incubated for 1 h at 37 °C. The supernatant (the medium) was then concentrated using an Amicon Ultra-15 Centrifugal Filter Unit (MilliporeSigma; catalog no.: UFC901024) with a 10 KDa cutoff by swinging bucket centrifugation at 4000*g* for 45 min at 4 °C. The resulting concentrated proteins were then prepared for Western blot analysis.

### Plasmids and siRNA

For knockdown experiments, cells were transfected with Silencer Select siRNAs (ThermoFisher Scientific; IDs s65969 [Fgf1 #2], s65971 [Fgf1 #1], and catalog no.: 4390843 [negative control siRNA]) at a final concentration of 1 pmol using Lipofectamine RNAiMAX Transfection Reagent (Invitrogen; catalog no.: 13778150) according to the manufacturer’s instructions. For overexpression experiments, cells were transfected with 100 ng Fgf1 (NM_010197) mouse-tagged ORF Clone (ORIGENE; catalog no.: MR201152) or control empty vector using Lipofectamine 3000 Transfection Reagent (Invitrogen; catalog no.: L3000001) according to the manufacturer’s instructions.

### Analysis of RNA-Sequencing Datasets

Analysis of mRNA was conducted using our previously published diurnal transcriptome datasets that were generated from the same cohort of mice as the proteomic data in this study or from a cohort of mice of identical age, sex, diet, and environmental conditions (Gene Expression Omnibus: GSE197726, GSE158600, and GSE197455) (5, 12). We used the differential rhythmicity and differential expression tool *dryR* (26) to identify rhythmic genes and genes with differential daily average expression between groups. *dryR* was performed on liver data from WT Alfp-Cre+ mice and *Bmal1* KO mice and on muscle data from WT Hsa-Cre+ mice and *Bmal1* KO mice. For the analysis, the starting set of genes oscillating in WT was defined as genes from rhythmic models 3, 4, and 5. Only genes with an mRNA expression value and a protein abundance value were analyzed.

### Gene Ontology Analysis

Pathway enrichment analysis was carried out using the Database for Annotation, Visualization, and Integrated Discovery software (27, 28). Enrichments for biological process, cellular compartment, and molecular function were used where indicated. *p* < 0.01 was considered statistically significant.

### Experimental Design and Statistical Rationale

All data are displayed as mean ± SEM unless otherwise noted. For each experiment, sample size, statistical tests, and significance threshold information can be found in the figure legend or main text. Sample sizes were determined by field standards. Four biological replicates were acquired for proteomics analysis, except for the TRF *Bmal1* KO group; one sample was determined to be an outlier according to heatmap and principal component analysis (PCA) clustering. This sample is highlighted in *red* in supplemental Table S1. For proteomics data, WT and total *Bmal1* KO mice are mice we consider as having complete clock function and complete loss of clock function, respectively. For experiments in cells, scrambled siRNAs or empty vectors served as controls. Complex statistical analyses of large-scale datasets are described within the corresponding Experimental Procedures section. Data were analyzed in Prism 6.0 software (GraphPad Software, Inc). The suitability of parametric *versus* nonparametric tests was determined by data distribution analysis tools in the software.

## Results and Discussion

### Tissue-Specific Reconstitution of *Bmal1* to Delineate Layers of Circadian Control of Liver and Muscle Proteomes

**Fig. 1:**
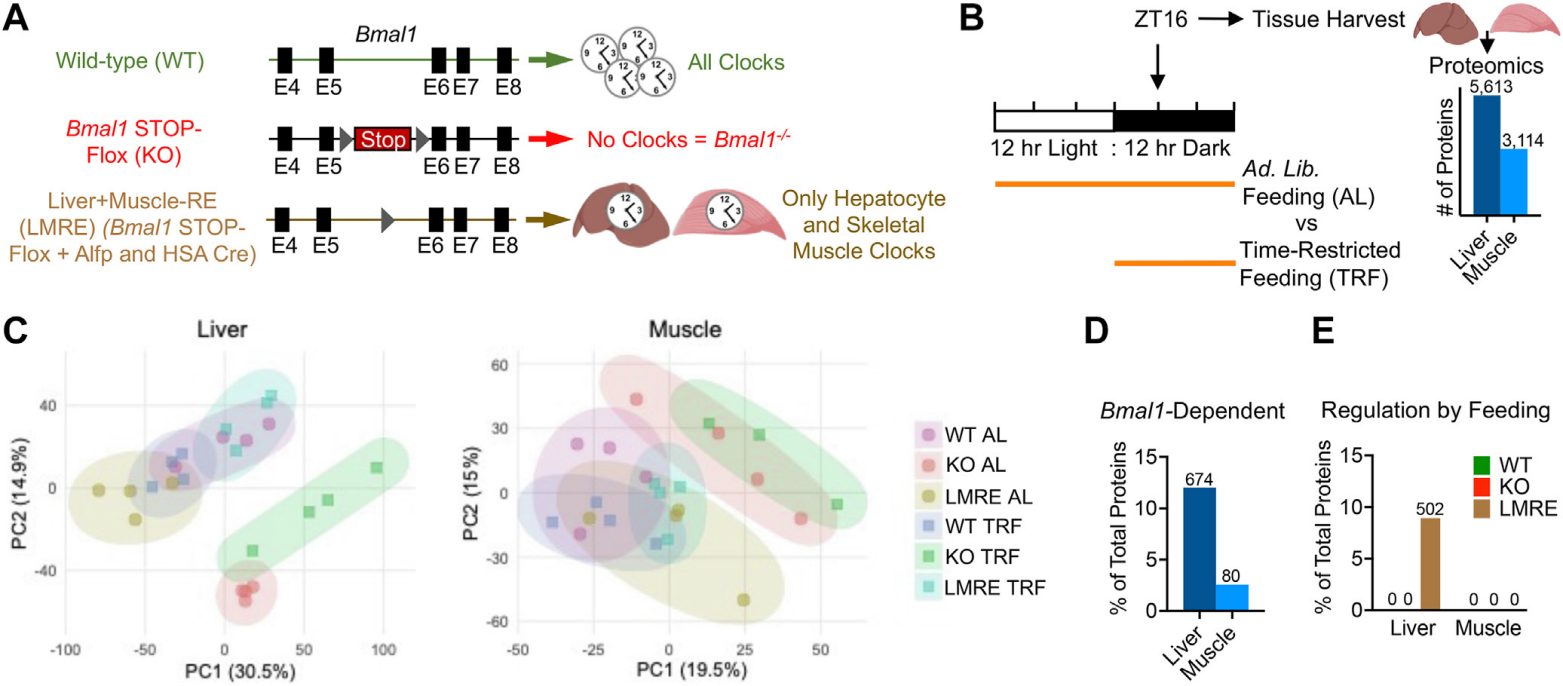
***Bmal1* remodels liver and skeletal muscle proteomes more potently than does time-restricted feeding (TRF).***A*, genetic scheme of cell type–specific reconstitution of the molecular circadian clock. *B*, overview of experimental design. Proteomic analysis by tandem mass tag (TMT) isobaric labeling combined with multidimensional liquid chromatography coupled to tandem mass spectrometry (n = 4). Values on bars are number of proteins quantified. *C*, principal component analysis (PCA) of tissue proteomes. *D* and *E*, Student’s *t* test with false discovery rate correction, *q* < 0.05, n = 4. Values on bars are number of proteins. *D*, proteins upregulated or downregulated in KO *versus* WT. *E*, proteins upregulated or downregulated in TRF *versus* AL within each genotype. Panels (*A* and *B*) created with BioRender.com. AL, *ad libitum*.

To define the regulation of tissue proteomes by *Bmal1* and/or daily feeding rhythms, we used *Bmal1*-stopFL mice, which do not express the main transcriptional activator of the molecular clock, *Bmal1*, except in Cre recombinase–expressing cells (4, 19) (Fig. 1*A*). *Bmal1*-stopFL mice lacking Cre (*Bmal1* KO) are analogous to conventional *Bmal1*-null mice and display severely impaired behavioral and molecular rhythms (4, 5, 19). Hepatocyte-specific Alfp-Cre and myonuclei-specific Hsa-Cre lines were crossed to generate a single line in which *Bmal1* was reconstituted (*i.e.*, rescued) in both liver and skeletal muscle (LMRE) (12). While this approach allowed us to analyze liver and muscle from the same mice, we acknowledge that the abundance of some proteins may be influenced by *Bmal1* function in the other tissue or by a synergistic effect of *Bmal1* in both tissues, rather than through rescue of local *Bmal1* function alone. We have previously shown that, as compared with WT, KO and LMRE mice exhibit no significant changes in food intake, yet have a lower body weight and a higher fat-to-lean mass ratio (12).

Because of lack of brain clock function (29), KO and LMRE mice lack robust 24 h rhythms of food intake under standard 12 h light/12 h dark conditions (supplemental Fig. S1*A*). To discern effects of physiological periods of feeding and fasting within and between genotypes, we placed mice under TRF, with access to food only during the 12 h dark active period (Fig. 1*B*). Thus, we imposed a daily feeding–fasting behavior on KO and LMRE mice (supplemental Fig. S1*A*), inducing WT-like daily rhythms of energy expenditure and a daily switch between carbohydrate and lipid oxidation (respiratory exchange ratio), with no discernable differences between KO and LMRE (12). We observed no significant changes in food intake in KO or LMRE mice under TRF, consistent with our previous reports (5, 12).

### *Bmal1* Remodels the Proteome More Extensively Than TRF

Liver and gastrocnemius muscle tissues were harvested from WT, KO, and LMRE mice at ZT16 (*i.e.*, 4 h into the dark phase), from mouse cohorts of AL feeding or TRF, giving rise to six experimental groups for each tissue (n = 4). This diurnal time point (ZT16) was identified as one of the peaks of rhythmic proteins in liver (16), at which point liver and muscle are engaged in the postprandial processing of macronutrients when mice are normally active and feeding. Proteomic analysis was performed using TMT isobaric labeling combined with multidimensional liquid chromatography coupled to tandem mass spectrometry. Each tissue was analyzed with three times TMT 10-plex experiments that included a pooled reference across each experiment for normalization. This analysis quantified 5613 proteins in liver and 3114 proteins in muscle, with coverage across diverse cellular compartments (Fig. 1*B*, supplemental Fig. S1*B*, and supplemental Table S1).

To determine the global impact of *Bmal1* and feeding cycles on tissue proteomes, we performed PCA. PCA plots revealed that liver proteomes segregated largely by the status of *Bmal1* and, to a much lesser extent, TRF (Fig. 1*C*). This was also true for muscle, yet the effect of *Bmal1* KO was comparatively less than in liver. In other words, the TRF that reinstated normal feeding behavior did not appear to rescue the liver or muscle proteome based on global PCA. This contrasts with LMRE proteomes, which partially clustered toward WT for each tissue.

We identified individual proteins affected by *Bmal1* by comparing WT to KO under AL conditions and then identified proteins regulated by feeding by comparing AL to TRF within each genotype (Student’s *t* test, Benjamini–Hochberg corrected *p* value [*q* value] <0.05) (supplemental Table S1). The potency of *Bmal1* over feeding rhythms was also reflected in this analysis; whereas loss of *Bmal1* affected 12.01% of detected liver proteins (Fig. 1*D*); the maximum effect of feeding in any genotype was 8.94% (Fig. 1*E*). In muscle, 2.57% of detected proteins were affected by *Bmal1*. Remarkably, however, TRF failed to change the abundance of any proteins in muscle, even considering a less stringent *q* value of <0.1. In liver, LMRE was the only genotype to respond substantially to TRF, with 8.94% of proteins affected. At *q* < 0.1, we observed 113 (2.01%) and 7 (0.13%) proteins affected by TRF in KO and WT mice, respectively. This disparity between number of regulated proteins in liver *versus* muscle remained when we also included proteins identified as *Bmal1* dependent under TRF (supplemental Fig. S1, *C* and *D*). As feeding rhythms strongly affect gene expression in both WT and clock mutant mice (11, 15, 29, 30), it was intriguing that TRF had a minimal impact at the proteome level. Yet the robust response to TRF in the liver of LMRE mice does provide evidence that *Bmal1* can modulate the proteomic response to feeding under certain conditions.

### Features of *Bmal1*-Dependent Proteomes in Liver and Skeletal Muscle

**Fig. 2:**
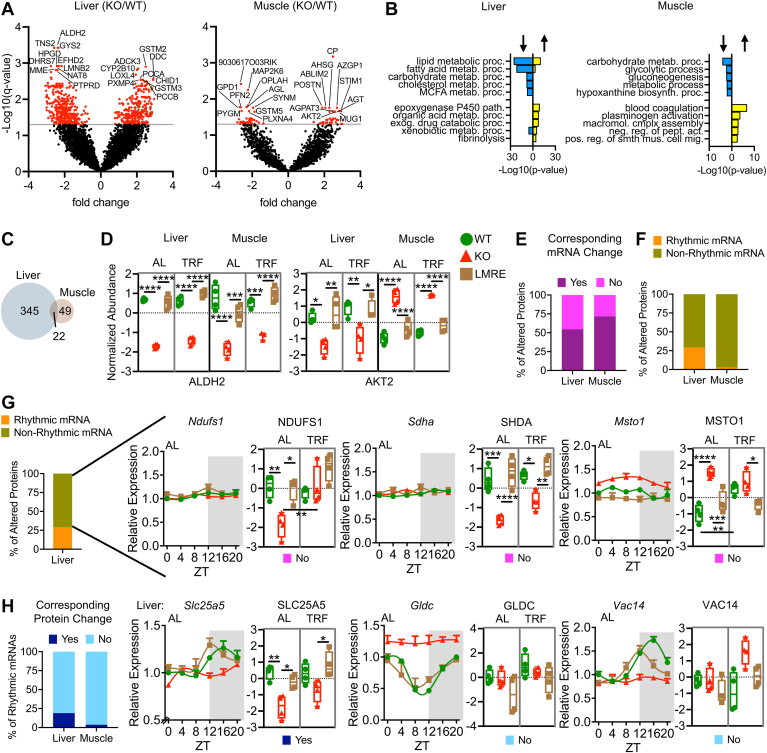
**Features of *Bmal1*-dependent proteomes in liver and skeletal muscle.***A*, volcano plots highlighting *Bmal1*-dependent proteins, Student’s *t* test with false discovery rate correction, *q* < 0.05, n = 4. Top 10 most significant upregulated and downregulated proteins are indicated. *B*, Gene Ontology enrichment (biological process) analysis of proteins upregulated (*right protruding bars*) and downregulated (*left protruding bars*) in KO *versus* WT. *C*, Venn diagram showing the overlap of *Bmal1*-dependent proteins in liver and muscle. Only proteins detected in both tissues were considered. *D*, examples of proteins regulated by *Bmal1* in both liver and muscle. Two-way ANOVA with Tukey’s multiple comparisons test, ∗*p* < 0.05, ∗∗*p* < 0.01, ∗∗∗*p* < 0.001, and ∗∗∗∗*p* < 0.0001. *E*–*H*, analysis, starting with *Bmal1*-dependent proteins, and interrogating their mRNA expression. WT and KO diurnal transcriptomes were analyzed with the differential rhythmicity algorithm *dryR*; rhythmic models were used to identify genes with rhythmic mRNA in WT, and mean models (difference in daily average expression) were used to identify genes with altered mRNA levels between WT and KO. See also supplemental Table S3. Statistics on protein plots—two-way ANOVA with Tukey’s multiple comparisons test, ∗*p* < 0.05, ∗∗*p* < 0.01, ∗∗∗*p* < 0.001, and ∗∗∗∗*p* < 0.0001, n = 4. *G*, examples of *Bmal1*-dependent liver proteins with nonrhythmic mRNA and no corresponding mRNA change in KO *versus* WT (*i.e.*, post-transcriptionally regulated). *Pink box* refers to groupings from *E*. *H*, examples of liver genes with rhythmic mRNA peaking near ZT16 and their corresponding protein abundances at ZT16. Of note, the protein abundances of GLDC and VAC14 break from their mRNA levels. All mRNA plots are normalized to WT ZT 0, n = 3 per time point per genotype. AKT2, RAC-beta serine/threonine-protein kinase; AL, *ad libitum* feeding; ALDH2, aldehyde dehydrogenase 2 mitochondrial; GLDC, glycine decarboxylase, mitochondrial; MSTO1, misato mitochondrial distribution and morphology regulator 1; NDUFS1, NADH:ubiquinone oxidoreductase core subunit S1; SDHA, succinate dehydrogenase complex, subunit A, flavoprotein; SLC25A5, solute carrier family 25, member 5; TRF, time-restricted feeding; VAC14, PIKFYVE complex component; ZT, zeitgeber time.

To visualize *Bmal1*-dependent changes in more detail, we generated volcano plots of all proteins and highlighted top hits (Fig. 2*A*). BMAL1, complexed with its binding partner CLOCK, is the main transcriptional activator of the molecular clock (31, 32). Hence, direct targets of BMAL1 are likely to be downregulated in its absence. Interestingly, we observed a nearly equal proportion of upregulated *versus* downregulated proteins in KO liver and muscle as compared with WT tissues. In line with the role of *Bmal1* in driving temporal metabolism (31), downregulated liver proteins were enriched for various lipid and carbohydrate pathways, and the top three downregulated muscle protein enrichments involved glucose (Fig. 2*B* and supplemental Table S2). Of note, liver and muscle shared upregulated pathways related to circulatory homeostasis, such as blood coagulation and fibrinolysis (Fig. 2*B*). Although most changes were indeed tissue specific, we identified 22 *Bmal1*-dependent proteins common to liver and muscle (Fig. 2*C*). For instance, aldehyde dehydrogenase mitochondrial (aldehyde dehydrogenase 2), an enzyme of alcohol metabolism, was reduced in KO and rescued in LMRE tissues, with no differences between AL and TRF conditions (Fig. 2*D* and supplemental Fig. S2*A*). Another interesting example was RAC-beta serine/threonine-protein kinase (AKT2), an enzyme of the insulin signal transduction pathway. AKT2 was upregulated in KO muscle but downregulated in KO liver (Fig. 2*D* and supplemental Fig. S2*A*). Such striking and disparate regulation likely stems from the tissue-specific outcomes of insulin signaling, with insulin inhibiting glucose production in liver and facilitating glucose uptake into muscle (33, 34).

Through proteomics analyses, we identified not only the products of many recognizable clock-controlled genes whose transcriptional rhythms are well documented but also novel targets whose regulation may or may not originate from changes in transcription. Many regulatory steps separate nascent transcription from steady-state protein abundance (*e.g.*, mRNA half-life, RNA processing events, translation efficiency). To tease apart whether the observed protein changes stemmed from changes in mRNA, we used *dryR* (26) mean models to identify genes whose average daily mRNA levels were differentially regulated between WT and *Bmal1* KO mice (supplemental Table S3). For this analysis, we used transcriptome data from our previous studies that featured identical experimental conditions yet with transcriptomic data obtained at six time points over the light–dark cycle (5, 12). By comparing average daily RNA abundance, we take into consideration that protein abundance may be slightly delayed with respect to RNA because of the added time frame of translation. The analysis revealed that 54.34% of affected liver proteins and 71.43% of affected muscle proteins had a corresponding change in average mRNA levels (Fig. 2*E*). These data implicate that post-transcriptional regulation has a larger role in shaping protein abundances in liver than muscle.

Next, we queried which *Bmal1*-regulated proteins at ZT16 were the product of rhythmic genes, which we defined using *dryR* rhythmic models of the WT transcriptomes of both tissues (supplemental Table S3). We found that most affected proteins were in fact the product of nonrhythmic genes (70.77% in liver and 97.14% in muscle; Fig. 2*F*). Focusing on liver, interrogation of functional enrichments unique to each group of proteins revealed that affected proteins of nonrhythmic genes were associated with mitochondrion organization and morphogenesis, among other pathways (Fig. 2*G* and supplemental Fig. S2, *B* and *C*). Notable examples were DNA polymerase subunit gamma-2 mitochondrial (POLG2), which promotes mitochondrial DNA synthesis, mitofusin-2, a GTPase essential for mitochondrial fusion, and many subunits of ubiquinone NADH dehydrogenase (NDUF, complex I), adding mechanistic insight to previous studies reporting a role for *Bmal1* in daily mitochondrial homeostasis (35). In muscle, *Dcaf7* was an example of a nonrhythmic gene with altered protein abundance (supplemental Fig. S2*D*).

Conversely, we identified genes with rhythmic mRNA and asked whether the abundance of their corresponding protein was altered by *Bmal1* KO. We included genes that peak within the dark phase (ZT14 to ZT22), which includes the ZT16 time point used for protein measurements. We found that only 19.01% of rhythmic liver genes and 3.70% of rhythmic muscle genes had proteins that were altered with loss of *Bmal1* (Fig. 2*H*). Unaffected proteins either represent genes whose rhythmicity is lost at the protein level or genes whose protein abundance lags behind mRNA abundance to a large extent. In liver, this group of unaffected proteins was uniquely enriched for Gene Ontology terms related to translation (supplemental Fig. S2*C*). In contrast, rhythmic genes that indeed had proteins that were altered by loss of *Bmal1* were uniquely enriched for pathways of energy metabolism (supplemental Fig. S2*C*). In muscle, *Glul* was identified as a rhythmic gene with highly correlated patterns of mRNA and protein abundance (supplemental Fig. S2*D*). Together, these data reveal that alterations in mRNA abundance upon loss of *Bmal1* may not be reflected in the protein abundance, and vice versa, underscoring a complex regulation of these aspects of cellular control by BMAL1.

### Partial Rescue of the Liver Proteome by Hepatocyte *Bmal1*

Next, we defined restoration of the liver proteome in LMRE mice. We had two possible explanations for proteins that were not rescued, which reflect organization of the clock network at the systemic and local levels: (1) hepatocyte *Bmal1* is insufficient to maintain the abundance of that protein, and extrinsic signals tied to *Bmal1* function elsewhere are needed and (2) the protein is primarily controlled by *Bmal1* in a different cell type. Considering that hepatocytes constitute the bulk of liver cells as well as liver protein (36), and that the expression of BMAL1 in LMRE liver is similar to WT (4, 5), we expected that the first explanation would apply in most cases. However, as nonparenchymal cells, such as endothelial cells, stellate cells, and Kupffer cells, contribute to the liver proteome (36, 37), the second explanation also has potential merit. As many functions are unique to hepatocytes, a functional analysis of proteins helped us to further tease apart the contribution of hepatocytes. PCA plots showed that AL proteomes of WT and LMRE livers were similar but did not overlap completely (Fig. 1*C*).

**Fig. 3:**
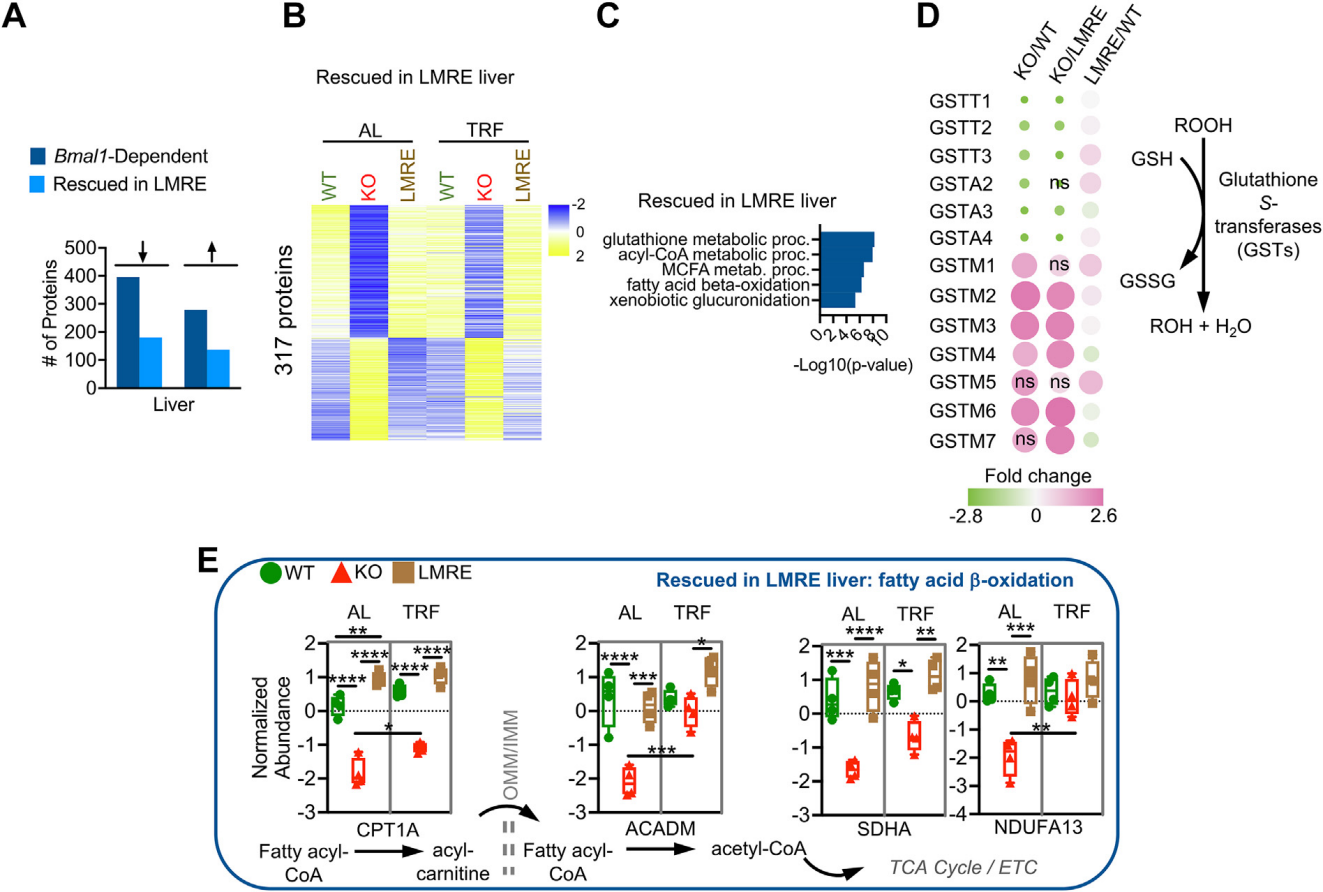
**Partial rescue of the liverproteome by hepatocyte *Bmal1*.***A*–*E*, *Bmal1*-dependent proteins (WT *versus* KO, *q* < 0.05) were statistically categorized by false discovery rate–corrected *p* values (*q* values) from Student’s *t* tests. Rescued in LMRE = WT *versus* KO, q < 0.05; LMRE *versus* KO *q* < 0.05; WT *versus* LMRE *q* > 0.05. *Arrows* indicate upregulation or downregulation in KO *versus* WT. *B*, heatmaps of rescued proteins. *C*, Gene Ontology enrichment (biological process) analysis showing the top five uniquely enriched pathways for each category of proteins. See also supplemental Table S2. *D*, bubble plot of quantified glutathione-*S*-transferases (GSTs) of the theta (T), alpha (A), and mu (M) classes. Bubble color and size are proportional to fold change. *E*, simplified scheme showing key steps of fatty acid β-oxidation. Two-way ANOVA with Tukey’s multiple comparisons test, ∗*p* < 0.05, ∗∗*p* < 0.01, ∗∗∗*p* < 0.001, ∗∗∗∗*p* < 0.0001, n = 4. ACADM, acyl-coenzyme A dehydrogenase, medium chain; CPT1A, carnitine palmitoyltransferase 1A, liver; ETC, electron transport chain; GSH, glutathione (reduced); GSSG, glutathione disulfide (oxidized); IMM, inner mitochondrial membrane; LMRE, liver and muscle reconstituted; MUFA, monounsaturated fatty acid; NDUFA13, NADH:ubiquinone oxidoreductase subunit A13; ns, not significant; OMM, outer mitochondrial membrane; ROOH, hydroperoxide functional group; SDHA, succinate dehydrogenase complex, subunit A, flavoprotein.

To identify the proteins responsible for this divergence, we assigned *Bmal1*-dependent proteins as rescued (*q* < 0.05, WT *versus* KO, LMRE *versus* KO; *q* > 0.05, WT *versus* LMRE) or nonrescued (*q* < 0.05, WT *versus* KO; *q* > 0.05, LMRE *versus* KO, WT *versus* LMRE) (Fig. 3, *A* and *B*, and supplemental Fig. S3*A*). We observed similar proportions of rescued (317) *versus* nonrescued (357) proteins, for both upregulated and downregulated proteins. Under TRF, many nonrescued proteins still appeared to be more similar between WT and LMRE liver (supplemental Fig. S3*A*). However, the effect of TRF was seemingly equal because of a reduction of protein abundance in WT mice and increased protein abundance in LMRE mice. Although TRF rescued certain proteins in KO tissues, protein abundance remained dysregulated overall (supplemental Fig. S3*A*).

Specific functions of rescued proteins were revealed by mapping proteins to pathways (Fig. 3*C*). Consistent with our previous studies (4, 5), rescued proteins were involved in functions such as NAD^+^ metabolism, involving the salvage pathway enzymes NAMPT and NMNAT3, and glycogen metabolism through GYS2, which are highly rhythmic processes in mouse liver. In line with the reasoning that nonrescued proteins are regulated by extrinsic signals, “response to nutrient” was among the top five enrichments of this category (supplemental Fig. S3*B*). We identified proteins involved in responding to xenobiotics (GCLC), energetic state (PKLR, GATM), specific energy substrates (HMGCL, AACS), and signaling activities (PTEN, adiponectin [ADIPOQ]) (supplemental Fig. S3*D*). Interestingly, top hits included enzymes involved in branched-chain amino acid catabolism (ACAD, HIBADH, IVD, and BCKDHB), a function intertwined with skeletal muscle (38) (supplemental Fig. S3*D*). Glucose transporter 2 was an example of partial rescue, showing a significant upregulation in LMRE liver *versus* KO under both AL and TRF conditions, yet not reaching WT abundance (supplemental Fig. S3*D*).

Several pathways were remarkably distinct in terms of rescue, thereby highlighting the intricacies of daily liver metabolism. Glutathione is a major antioxidant that detoxifies reactive oxygen species, xenobiotics, and peroxides. While GCLC, the rate-limiting enzyme for glutathione synthesis, was not rescued in LMRE (supplemental Fig. S3*D*), nearly all glutathione-*S*-transferases (GSTs) were (Fig. 3*D*). GSTs use glutathione to reduce, and in effect detoxify, diverse substrates (39). We observed full rescue of the abundance of GST proteins belonging to the alpha, mu, and theta classes. Intriguingly, only mu proteins were upregulated in *Bmal1* KO livers (Fig. 3*D*), indicating dichotomy in the directionality of regulation among GSTs, which may relate to substrate specificity. Glutathione rhythms are reported to anticipate their need, peaking just prior to the intake of potentially harmful substances during feeding at the onset of nighttime (40, 41). Our data showed that GST abundances were unaffected by TRF (supplemental Fig. S3*E*). Together, these data are indicative of extrinsic control of the machinery for glutathione production and intrinsic clock control of GSTs, helping to explain the roles of the liver clock and feeding rhythms in controlling chronotoxicity in the liver (42, 43).

“Lipid metabolic process” was the top enrichment category for both rescued and nonrescued proteins (supplemental Table S2), but a more detailed analysis of this large pathway revealed that fatty acid oxidation was enriched in rescued proteins, whereas the opposing process, fatty acid synthesis, was enriched in nonrescued proteins (Fig. 3*C*, supplemental Figs. S3*B*, and S4*A*). The liver is a major hub in organismal lipid metabolism. Fatty acids are oxidized as energy substrates, synthesized from carbohydrate building blocks, stored, or circulated to other tissues in the form of triglycerides. These activities exhibit circadian rhythms to anticipate opportunities to store energy during feeding (*de novo* lipogenesis) and utilize energy stores during periods of fasting (fatty acid β-oxidation) (6, 44, 45). Enzymes mediating key steps in peroxisomal and mitochondrial fatty acid oxidation were rescued in LMRE livers (Fig. 3, *C* and *E*), including carnitine palmitoyltransferase 1a, which catalyzes the rate-limiting step for long-chain fatty acids, and acyl-coenzyme, a dehydrogenase medium chain, which acts on medium-chain fatty acids (supplemental Fig. S3*C*). Mitochondrial proteins that support energy production downstream of β-oxidation were also numerous among fully rescued proteins. In contrast, enzymes mediating key steps in *de novo* lipogenesis were classified as nonrescued (supplemental Figs. S3*B* and S4*A*). The rate-limiting step in *de novo* lipogenesis is the carboxylation of acetyl-CoA to malonyl-CoA by acetyl-CoA carboxylase (ACC). ACC alpha abundance was similarly downregulated in KO and LMRE livers under AL conditions. This was also true for ATP citrate synthase, which produces acetyl-CoA upstream of ACC, and for fatty acid synthase, which synthesizes palmitate downstream of ACC (supplemental Fig. S4*A*). These changes were concomitant with the lower abundance of sterol regulatory element–binding protein 1, a transcription factor that promotes lipogenesis (46) (supplemental Fig. S4*B*). As these data were acquired at ZT16, a time point characterized by higher *de novo* lipogenesis activity (6, 46), the protein abundances demonstrate that extrinsic signals support fatty acid–synthesizing machinery. Our data show that one potential extrinsic signal—timing of feeding—tended to normalize protein abundance among the genotypes, but this effect was partially driven by a reduction in protein abundance in WT. These results highlight the importance of nonautonomous regulation by the clock system on hepatic lipogenesis.

### Partial Rescue of the Muscle Proteome by Myofiber *Bmal1*

Next, we defined the ability of *Bmal1* in muscle fibers to rescue the skeletal muscle proteome. Recent single-nucleus sequencing studies show that ∼70% of nuclei within skeletal muscle are the muscle fiber–associated myonuclei (47). Here, we used Hsa-Cre to rescue *Bmal1* specifically in such myonuclei. Muscle fibers are primary sites of metabolic activity in the muscle, and we have previously shown that *Hsa*-Cre recombination is highly efficient in LMRE muscles (12). Accordingly, our model is suitable for investigating proteomic responses of these cells. Like liver, nonrescued proteins in muscle may be produced by nonmyolineage cell types or be regulated by missing extrinsic signals from other tissue clocks.

**Fig. 4:**
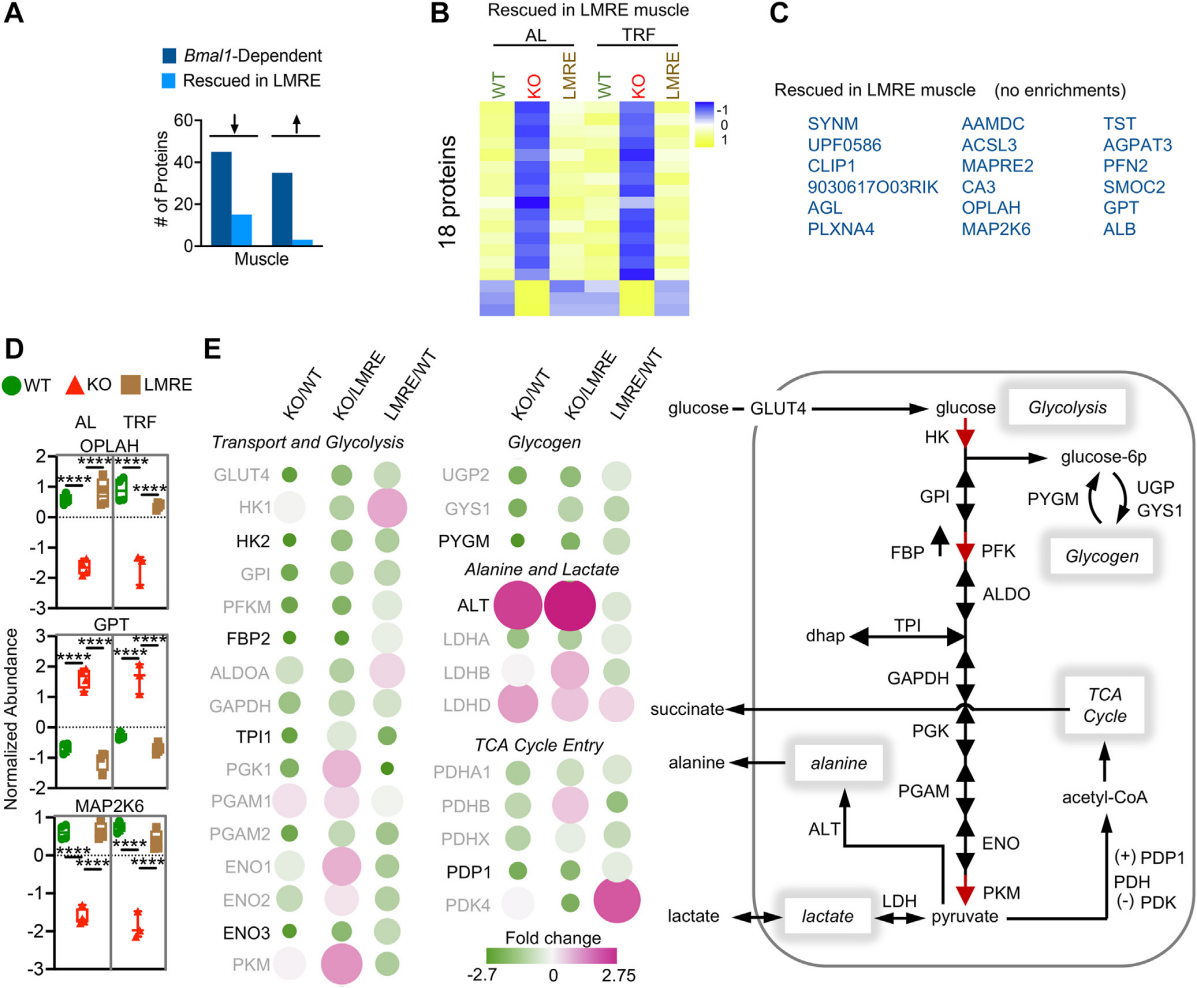
**Partial rescue of the skeletal muscleproteome by myofiber *Bmal1*.***A*–*E*, *Bmal1*-dependent proteins (WT *versus* KO, *q* < 0.05) were statistically categorized by false discovery rate–corrected *p* values (*q* values) from Student’s *t* tests. Rescued in LMRE = WT *versus* KO, *q* < 0.05; LMRE *versus* KO *q* < 0.05; WT *versus* LMRE *q* > 0.05. *Arrows* indicate upregulation or downregulation in KO *versus* WT. *B*, heatmaps of rescued proteins. *C*, Gene Ontology enrichment (biological process) analysis showing the top five uniquely enriched pathways for each category of proteins. See also supplemental Table S2. *D*, examples of rescued proteins in muscle of LMRE mice. Two-way ANOVA with Tukey’s multiple comparisons test, ∗∗∗∗*p* < 0.0001, n = 3 to 4. *E*, *left*, bubble plot of quantified proteins that carry out key steps in glucose metabolism. Bubble color and size are proportional to fold change. Nonsignificant changes have a grayed-out protein name. *Right*, simplified scheme showing key steps in muscle glucose metabolism. ALDOA, aldolase A; ALT (GPT), alanine aminotransferase 1; ENO, enolase; DHAP, dihydroxyacetone phosphate; FBP2, fructose bisphosphate 2; GLUT4 (SLC2A4), solute carrier family 2 member 4; GPI, glucose-6-phosphate isomerase; GYS1, glycogen synthase 1; HK, hexokinase; LDH, lactate dehydrogenase; LMRE, liver and muscle reconstituted; MAP2K6, mitogen-activated protein kinase kinase 6; OPLAH, 5-oxoprolinase; 6p, 6-phosphate; PDH, pyruvate dehydrogenase; PDK4, pyruvate dehydrogenase kinase, isoenzyme 4; PDP1, pyruvate dehydrogenase phosphatase catalytic subunit 1; PFKM, phosphofructokinase, muscle; PGAM, phosphoglycerate mutase 1; PGK1, phosphoglycerate kinase 1; PKM, pyruvate kinase, muscle; PYGM, muscle glycogen phosphorylase; TPI1, triosephosphate isomerase 1; UGP2, UDP-glucose pyrophosphorylase 2.

We statistically categorized muscle proteins as rescued or nonrescued and visualized the results with heatmaps (Fig. 4, *A* and *B* and supplemental Fig. S5*A*). About 15 of the 18 proteins rescued in LMRE were downregulated in KO muscle as compared with WT muscle. This overall directionality in effect was not observed in liver or for nonrescued proteins in muscle. In line with the small number of proteins identified in this classification, we did not observe any significant functional enrichments for rescued proteins (Fig. 4*C*). Notable proteins included glutamic pyruvic transaminase (also known as alanine aminotransferase 1), which serves a key function in muscle carbohydrate metabolism by converting glycolysis-derived pyruvate to alanine; 5-oxoprolinase, an ATP-hydrolyzing enzyme involved in glutamate production; and mitogen-activated protein kinase kinase 6, a regulator of mitogen-activated protein kinase (Fig. 4, *C* and *D* and supplemental Fig. S5*B*).

Two of the top five functional enrichments of nonrescued proteins involved glucose metabolism (supplemental Fig. S4*C*). During feeding, most glucose is cleared from the blood *via* insulin-induced uptake into skeletal muscle (48, 49). This process is under circadian control: the central clock in the brain drives mice to consume the most food during their active dark phase (50), BMAL1 modulates glucose-stimulated insulin release from pancreatic beta cells (8), and muscle BMAL1 times the expression of key enzymes to meet the demand (51). By tracing ^13^C-labeled glucose in LMRE mice, we have recently shown that muscle BMAL1 is necessary but not sufficient for maximal glucose uptake and oxidation (12), and that the addition of a liver clock in AL conditions does not augment this function. In line with this finding, we observed that protein abundance for several key glucose enzymes was still deregulated in the muscle of LMRE mice (Fig. 4*E*).

Glucose has several fates in muscle: (1) oxidation *via* the tricarboxylic acid (TCA) cycle, (2) conversion to lactate or alanine as a glycolytic endpoint, (3) storage as glycogen, and (4) release as succinate (48, 52, 53, 54) (Fig. 4*E*). Oxidation supports energy production, glycogen supplies glycolytic intermediates (and substrates for energy production), and lactate and alanine provide the liver with substrates for the TCA cycle, gluconeogenesis, and the disposal of nitrogen. Several glycolysis enzymes, including the rate-limiting enzyme hexokinase 2, showed a trend for partial restoration in LMRE muscles (Fig. 4*E*). The muscle isoform of the primary glycogenolysis enzyme, glycogen phosphorylase muscle associated (PYGM), also appeared to be partially rescued, along with pyruvate dehydrogenase phosphatase catalytic subunit 1 (Fig. 4*E*). Pyruvate dehydrogenase phosphatase catalytic subunit 1 dephosphorylates and activates the pyruvate dehydrogenase complex, generating acetyl-CoA that feeds into the TCA cycle. These data suggest that the abundance of enzymes responsible for processing glucose and directing the flow of its carbons is more strongly supported by muscle-extrinsic functions of *Bmal1*. Because their abundance still showed tendencies for improvement as compared with KO muscle, we posit that the muscle-extrinsic functions of BMAL1 fine-tune or provide robustness to these pathways.

### An Interaction Between *Bmal1* and Feeding Dictates Ribosomal Protein Abundances in Liver

**Fig. 5:**
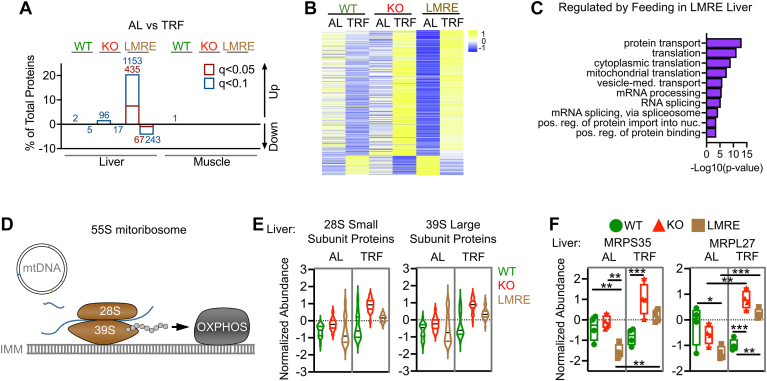
**An interaction between *Bmal1* and feeding rhythms dictates ribosomal protein abundances in liver.***A*, effect of feeding on protein abundances within genotypes. Student’s *t* test with false discovery rate correction, *q* < 0.05, n = 4. Results using a less-stringent *q* value (0.1) are shown for comparison. Values on bars are number of proteins. *B* and *C*, analysis of liver proteins affected by TRF (*q* < 0.05) in LMRE. *B*, heatmap. *C*, Gene Ontology enrichment (biological process) analysis showing the top 10 enriched pathways. *D*, simplified scheme of the mitoribosome and its function in the mitochondrion. The small (28S) and large (39S) subunits form the 55S mitoribosome that translates RNA produced from mitochondrial DNA (mtDNA). RNAs include those that encode proteins of the complexes of the electron transport chain, which enables oxidative phosphorylation (OXPHOS). *E*, violin plot showing the average normalized abundance of 27 small subunits (28S, *left*) and 45 large subunits (39S, *right*) in liver. *F*, example abundances of small and large mitoribosome subunits in liver. Two-way ANOVA with Tukey’s multiple comparisons test, ∗*p* < 0.05, ∗∗*p* < 0.01, ∗∗∗*p* < 0.001, n = 4. AL, *ad libitum* feeding; IMM, inner mitochondrial membrane; LMRE, liver and muscle reconstituted; MRPL27, mitochondrial ribosomal protein L27; MRPS35, mitochondrial ribosomal protein S35; TRF, time-restricted feeding.

TRF paradigms substantially modulate mRNAs, especially in clock mutant mice that lack a typical feeding–fasting rhythm under AL conditions (11, 15, 29, 30); however, changes in protein abundances in response to TRF are less well characterized. When comparing AL *versus* TRF within genotypes, we observed that LMRE was the only genotype to respond robustly to TRF (502 proteins, *q* < 0.05), and this only occurred in liver (Fig. 5*A*). There were no proteins affected in WT or KO at this significance threshold. Trends were maintained if the threshold was eased to *q* < 0.1, with muscle still lacking any feeding-responsive proteins, irrespective of genotype. This global analysis indicates that at the protein level at ZT16, the liver is a target of TRF as compared with muscle.

A heatmap of the 502 feeding-responsive proteins from LMRE livers revealed interesting patterns of protein abundance between the genotypes (Fig. 5*B*). In KO and LMRE, most proteins appeared upregulated by TRF, but changes were only significant in LMRE, and changes in KO were considerably smaller in magnitude. Conversely, the same proteins were modesty and nonsignificantly downregulated by TRF in WT. WT mice already enact a robust feeding–fasting rhythm, and we reveal that an enhancement of this behavior by TRF had only a minimal impact, even if thousands of genes at the transcript level are affected by the same TRF paradigm in WT mice (5). Similarly, TRF can positively impact the transcriptome of *Bmal1* KO livers (15, 29). However, these current data indicate quite different protein-level responses to TRF at ZT16, suggesting a buffering capacity of circadian genes at the protein level similar to that suggested for genes expressed in a bidirectional manner (55). From these results, we concluded that liver *Bmal1* and extrahepatic *Bmal1* augment the liver’s response to TRF.

We identified three main cellular activities that dominated the feeding-responsive proteins in liver: protein transport, translation, and RNA processing (Fig. 5*C*). Previous studies have elucidated mechanisms by which translation is controlled by the clock and feeding (13, 14, 56). In mouse livers, a subset of mRNAs displays increased translation during the dark phase, facilitated by increased ribosome biogenesis that is aligned with feeding time (14). On one hand, the molecular clock shapes this rhythm by controlling transcription of translation initiation factors, ribosomal proteins, and ribosomal RNAs (56). On the other hand, daily feeding rhythms drive nutrient-responsive signaling pathways, and nutrient status, in turn, is a potent regulator of translation (13). Here, considering translation machinery and regulatory proteins under AL conditions, we did not observe any enriched functions related to translation in liver of *Bmal1* KO mice (supplemental Table S2). Instead, enrichment of translation Gene Ontology terms was specific to conditions in which feeding–fasting cycles were engaged. A more detailed analysis revealed that only one ribosomal protein (RPL13) was affected by complete loss of *Bmal1* (under AL feeding), whereas 62 proteins were regulated by feeding (supplemental Fig. S6*A*). Proteins constituting both the large (60S) and small (40S) ribosomal subunits were affected. Several members of the eukaryotic initiation factor protein family were upregulated by TRF in LMRE. These increased protein abundances may indicate increased translation, and our analysis was performed in a diurnal phase associated with maximal translation, ribosome accumulation, and typical feeding times. Therefore, our data suggest that circadian control of feeding behavior is an important driver of translation in liver.

Analysis of feeding-responsive proteins also illuminated a previously unappreciated effect on mitochondrial translation. In addition to cytoplasmic ribosomes, a distinct set of ribosomal proteins constitutes two ribosome subunits assembled within the mitochondria (57) (Fig. 5*D*). One 28S small subunit and one 39S large subunit comprise a mitochondrial ribosome (the 55S mitoribosome). We found that average expression levels of 28S and 39S mitochondrial ribosomal proteins exhibit a similar pattern of abundance as the individual proteins we identified as statistical hits (Fig. 5, *E* and *F*, and supplemental Fig. S6*B*). The constituent ribosomal proteins are encoded by nuclear DNA and support translation of proteins encoded by the mitochondrial DNA. This process is critical for proper oxidative phosphorylation, as the protein-coding genes of mitochondrial DNA produce subunits of complex I, IV, and V of the electron transport chain (58). The fact that these changes were only observed in LMRE suggests that local clocks transduce signals from feeding to regulate mitochondrial translation.

### Secreted Proteins are Under BMAL1 Control in Liver and Muscle

Liver and muscle cells also produce extracellular or secreted proteins. Secreted proteins modulate a wide range of functions through autocrine, paracrine, and endocrine mechanisms. The timing of our proteomics analysis corresponded with theorized time of accumulation of secreted proteins within the liver (ZT16, nighttime) (16). Therefore, we used the opportunity to identify secreted proteins regulated by *Bmal1* and TRF.

**Fig. 6:**
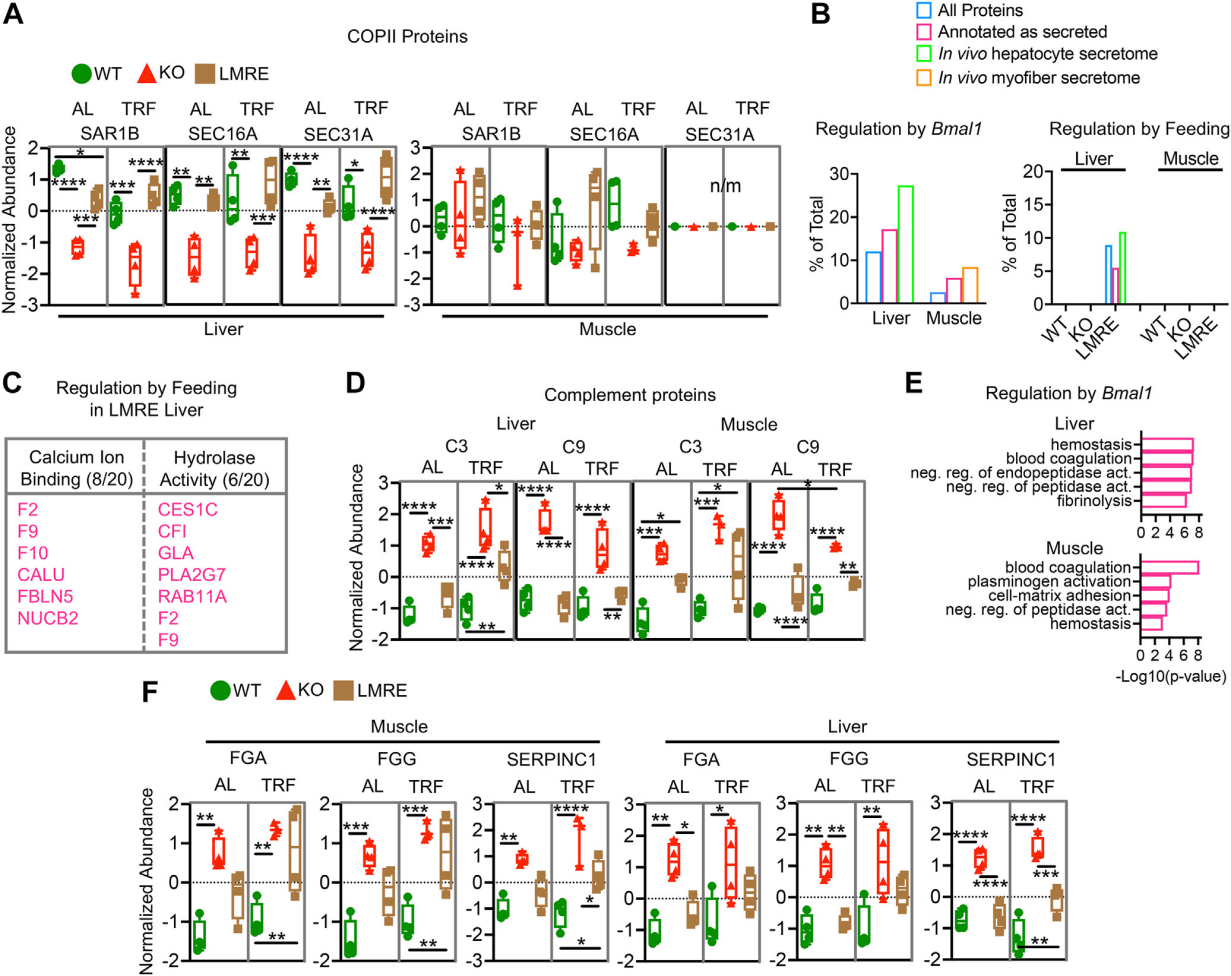
**Secreted proteins are targeted by *Bmal1* in liver and skeletal muscle.***A*, example proteins from the coat protein 2 (COPII) complex, a mechanism of secreted proteins that enables the formation of vesicles to transport proteins from the endoplasmic reticulum to the Golgi apparatus. Two-way ANOVA with Tukey’s multiple comparisons test, ∗*p* < 0.05, ∗∗*p* < 0.01, ∗∗∗*p* < 0.001, and ∗∗∗∗*p* < 0.0001, n = 4 (n = 3 for TRF KO muscle). *B*–*F*, analysis of secreted proteins. *B*, Student’s *t* test with false discovery rate correction, *q* < 0.05, n = 4, WT *versus* KO (regulation by *Bmal1*), AL *versus* TRF within genotype (regulation by feeding). Displayed as percentage of detected proteins in that class. Secreted proteins either annotated as secreted *via* UniProt or published *in vivo* secretomes for hepatocytes or myofibers (61). *C*, Gene Ontology enrichment (molecular function) analysis of secreted proteins regulated by feeding in LMRE liver. Numbers indicate hits/total proteins in class. *D*, analysis of complement proteins in liver and muscle. Two-way ANOVA with Tukey’s multiple comparisons test, ∗*p* < 0.05, ∗∗*p* < 0.01, ∗∗∗*p* < 0.001, and ∗∗∗∗*p* < 0.0001, n = 4. *E*, Gene Ontology enrichment analysis showing the top five enriched pathways. *F*, analysis of coagulation cascade proteins in liver and muscle. Two-way ANOVA with Tukey’s multiple comparisons test, ∗*p* < 0.05, ∗∗*p* < 0.01, ∗∗∗*p* < 0.001, and ∗∗∗∗*p* < 0.0001, n = 4. AL, *ad libitum* feeding; C3, complement component 3; C9, complement component 9; CALU, calumenin; CES1C, carboxylesterase 1C; CFI, complement component factor I; F, coagulation factor; FBLN5, fibulin 5; FGA/G, fibrinogen alpha and gamma chains; GLA, galactosidase, alpha; LMRE, liver and muscle reconstituted; n/m, not measured; NUCB2, nucleobindin 2; PLA2G7, phospholipase A2; RAB11A, member RAS oncogene family; SAR1B, secretion-associated Ras-related GTPase 1B; SEC16A, SEC16 homolog A, endoplasmic reticulum export factor; SEC31A, SEC31 homolog A; SERPINC1, serine protease inhibitor c 1; TRF, time-restricted feeding.

Most proteins are secreted *via* the classical/conventional secretory pathway—synthesized by endoplasmic reticulum (ER)–tethered ribosomes, transferred in vesicles to the Golgi apparatus, packaged into secretory vesicles, and trafficked to the plasma membrane for release *via* exocytosis (59, 60). Proteins downregulated in *Bmal1* KO livers were enriched for ER to Golgi vesicle–mediated transport, lipoprotein transport, and vesicle-mediated transport (supplemental Table S2), suggesting that BMAL1 supports protein secretion. Several components of coat protein complex II, which facilitates the formation of vesicles for ER to Golgi transport, were downregulated in *Bmal1* KO and rescued in LMRE (including secretion-associated Ras-related GTPase 1b; SEC16 and 31 homolog A) (Fig. 6*A* and supplemental Fig. S7*A*), as were components of coat protein complex I, which mediates retrograde Golgi to ER transport. These changes were not observed in muscle (Fig. 6*A*).

To interrogate secreted proteins from our dataset, we either filtered for “extracellular” Gene Ontology classification or used published *in vivo* secretomes for hepatocytes and myofibers (61). In both cases, we found that secreted proteins were more heavily affected by *Bmal1* KO than the total protein pool, suggesting an importance of this group of proteins among clock-controlled genes (Fig. 6*B*). LMRE liver was the only group to exhibit changes in secreted proteins in response to TRF (Fig. 6*B*). Of the 30 feeding-responsive proteins annotated as secreted in LMRE, 10 were also modulated by BMAL1. Among the 20 feeding-responsive proteins were many zymogens and proenzymes, inactive precursor peptides that require further cleavage to become active (Fig. 6*C*). Zymogens are key steps in blood coagulation and complement (immune system) cascades (62). The thrombin precursor prothrombin (F2), the plasmin precursor plasminogen, the kinin precursor kininogen 2, and the coagulation factors IX and X (F9 and F10) were upregulated by TRF in LMRE livers. The same trend was observed in KO livers, but increases were not statistically significant, implying that *Bmal1* is required to enhance the responses of these proteins to feeding.

Notably, complement cascade proteins were strongly upregulated in both liver and skeletal muscle of *Bmal1* KO mice (Fig. 6*D*). Of the 23 detected complement proteins, 9 (39%) were regulated by BMAL1 in at least one tissue, and the abundance of many was restored in LMRE mice. Coagulation cascade proteins were also regulated by BMAL1 in both tissues (Fig. 6*E*). This group included fibrinogen alpha and gamma chains and regulators of protease activity including serine protease inhibitor C1 (SERPINC1, also known as antithrombin-3) (Fig. 6*F* and supplemental Fig. S7*A*). These pathways are regulated by the serpin family of serine protease inhibitors (63, 64). In addition to SERPINC1, five other serpin family members were upregulated in *Bmal1* KO livers (SERPIN -A6, -B6B, -A1E, -F1, and -B1A). Interleukins 6 and 8 and chemokine c–c motif ligand 2 (also called MCP-1) are rhythmically released from human skeletal myotubes synchronized *in vitro* (65). However, these proteins were not detected in our dataset, perhaps because of their relatively low abundance in muscle prior to stimulation by exercise or circulating immune cells (66).

The number of secreted proteins affected by loss of *Bmal1* was more extensive in liver than muscle. Liver-secreted proteins are enzymes, plasma proteins (*e.g.*, albumin), hemostasis factors, apolipoproteins, components of the extracellular matrix, growth factors, and hormones (59, 67). They contribute substantially to the makeup of the serum proteome (68, 69). Metabolic pathways were among the functional enrichments of liver *Bmal1*-dependent secreted proteins, specifically cholesterol and lipid metabolism (supplemental Table S2). We observed that the abundance of hepatic lipase (lipase C [LIPC]), the primary enzyme that catalyzes the hydrolysis of triglycerides to diacylglycerol and free fatty acids, was upregulated twofold in KO, rescued to WT levels in LMRE, and remarkably stable under TRF (supplemental Fig. S7, *B* and *C*). LIPC can exist within the liver bound to local endothelial cells or enter the circulation in complex with lipoproteins. It plays an important role in setting organismal levels of high-density lipoprotein and low-density lipoprotein (LDL) cholesterols (70, 71). Upregulation of LIPC was accompanied by downregulation of the LDL receptor and its transcriptional regulator sterol regulatory element–binding protein 1 (46, 72) (supplemental Figs. S7*C* and S4*B*). This regulation of lipoprotein machinery likely contributes to the lipid phenotypes observed in clock mutant mice (11, 44, 73, 74, 75). Interestingly, TRF tended to normalize liver abundance of sterol regulatory element–binding protein 1 and LDL receptor to WT levels.

**Fig. 7:**
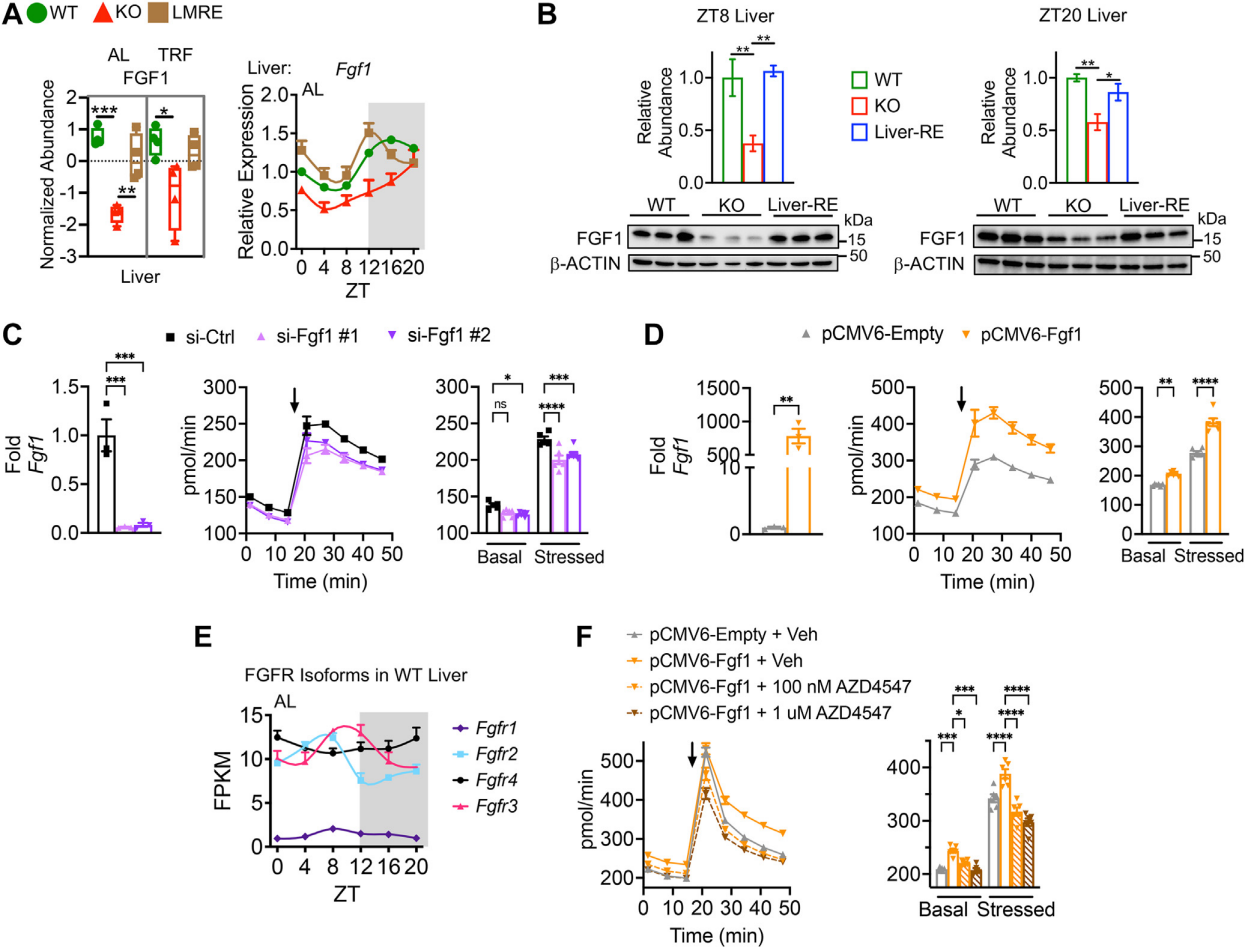
***Bmal1* regulates FGF1, which supports mitochondrial respiration in hepatocytes.***A*, mRNA (*right*) and protein abundance (*left*) of FGF1 protein in liver. Two-way ANOVA with Tukey’s multiple comparisons test, ∗*p* < 0.05, ∗∗*p* < 0.01, ∗∗∗*p* < 0.001, and ∗∗∗∗*p* < 0.0001, n = 4. *B*, Western blot for FGF1 in liver at the indicated diurnal time points. Biological replicates are shown (*bottom*) and quantified (*top*). One-way ANOVA with Fisher’s LSD test, ∗*p* < 0.05 and ∗∗*p* < 0.01, n = 3. Liver-RE = *Bmal1*^*stopFL/stopFL*^; *Alfp*-Cre^tg/0^, hepatocyte-specific reconstitution of *Bmal1*. *C* and *D*, knockdown or overexpression of *Fgf1* in AML12 hepatocytes. si-Ctrl = scrambled sequence; both si-Fgf1 #1 and #2 target *Fgf1* but by different sequences. pCMV6-Empty = control vector without *Fgf1* open reading frame clone. *Left*, qPCR, Student’s *t* test, ∗∗*p* < 0.01 and ∗∗∗*p* < 0.001, n = 3. *Middle*, oxygen consumption rate in AML12 hepatocytes. *Arrow* indicates application of 0.5 μM carbonyl cyanide-*p*-trifluoromethoxyphenylhydrazone (FCCP) and 1 μM oligomycin. *Right*, quantification of *middle*. Basal and stressed values are averages of data points before and after drug application, respectively. Two-way ANOVA with Dunnett’s post hoc test, ∗*p* < 0.05, ∗∗*p* < 0.01, ∗∗∗*p* < 0.001, and ∗∗∗∗*p* < 0.0001, n = 5 to 6. *E*, mRNA levels of FGF receptor (FGFR) isoforms in WT (Alfp-Cre+ and Hsa-Cre+) liver. *F*, measurement of oxygen consumption rate as in *D* and *E*. AML12 cells were treated for 24 h with vehicle control (Veh) or the indicated concentration of the pan-FGFR inhibitor AZD4547. Two-way ANOVA with Tukey’s post hoc test, ∗*p* < 0.05, ∗∗∗*p* < 0.001, and ∗∗∗∗*p* < 0.0001, n = 5 to 6. All mRNA plots are normalized to WT ZT 0, n = 3 per time point per genotype. AL, *ad libitum* feeding; AML12, alpha mouse liver 12; FGF, fibroblast growth factor; LSD, least significant difference; ns, not significant; qPCR, quantitative PCR; TRF, time-restricted feeding; ZT, zeitgeber time.

Particularly, noteworthy changes in *Bmal1* KO liver were a decrease in FGF1 (Fig. 7*A*), a metabolic regulator, and an increase in ADIPOQ (supplemental Fig. S3*D*). A similar but nonsignificant trend was also observed for ADIPOQ in muscle (supplemental Fig. S3*D*). Adiponectin is an endocrine hormone produced by adipose tissue that regulates glucose and fatty acid metabolism in target tissues, including liver (76). Its activity is associated with the suppression of metabolic impairments related to type 2 diabetes and fatty liver disease (77, 78). There is evidence that ADIPOQ levels are intertwined with feeding and fasting behavior (79); however, we did not observe a substantial change in ADIPOQ levels from AL to TRF in any genotype, suggesting a more prominent regulation by *Bmal1*.

### Bmal1 Regulates FGF1 in Liver

FGF1 (also known as acidic FGF) was originally identified as an endothelial growth factor (*i.e.*, mitogen) but has reemerged as a metabolic regulator (80). When challenged with a Western-style diet or genetically induced obesity, mice injected with recombinant FGF1 protein exhibit substantial rescue of hepatic steatosis and hepatic insulin sensitivity as well as suppression of hepatic glucose production (81, 82). Despite these remarkable effects of FGF1 signaling on liver metabolism, the functional significance of the endogenous FGF1 protein in liver remains obscure.

Of the 22 FGF proteins, only FGF1 was quantified in our liver dataset, suggesting it is the most highly expressed isoform (supplemental Table S1 and Fig. 7*A*). Indeed, *Fgf1* exhibited the highest expression at the RNA level, with ∼10-fold higher expression than *Fgf21* (supplemental Fig. S8*A*). In addition, *Fgf1* exhibited small amplitude circadian variation, with peak expression during nighttime (Fig. 7*A* and supplemental Fig. S8*A*). Next, we confirmed *Bmal1*-dependent regulation of FGF1 by Western blot. FGF1 was significantly reduced in KO compared with WT livers harvested either during the rest phase (ZT8) or the active phase (ZT20), and rescue of only hepatocyte *Bmal1* (liver-RE mice) was sufficient to restore liver FGF1 abundance (Fig. 7*B*). Although *Fgf1* mRNA displayed a peak during the dark phase, we did not detect any changes at the protein level in our analysis of two time points (supplemental Fig. S8*B*). Because rescue of hepatocyte *Bmal1* rescued FGF1 abundance, our data indicate that hepatocytes are the main producers of FGF1 protein in liver. AML12 cells, a nontransformed hepatocyte cell line, also expressed FGF1 protein (supplemental Fig. S8*C*).

### Fgf1 Supports Mitochondrial Respiration in Hepatocytes

A common thread among the reported effects of FGF1 on liver is on mitochondrial function (80, 81, 82). We hypothesized that FGF1 could have a substantial impact on metabolism through the regulation of mitochondrial respiration, a process critical for catabolic and anabolic pathways in hepatocytes. To test this idea, we knocked down or overexpressed *Fgf1* in AML12 hepatocytes by siRNA and a mammalian expression vector, respectively. We confirmed efficient knockdown and overexpression by qPCR (Fig. 7, *C* and *D*) and then measured oxygen consumption, a readout of mitochondrial respiration, under basal or stressed conditions. Stressed conditions were created by adding the uncoupling agent carbonyl cyanide-*p*-trifluoromethoxyphenylhydrazone and the ATP synthase inhibitor oligomycin. Because FGF1 regulates the cell growth, we plated confluent cells and transfected them at the time of plating (reverse transfection). With this design, the number of cells was kept constant across the groups, so that it did not confound measurements. We found that *Fgf1* knockdown lowered oxygen consumption, and conversely, its overexpression increased oxygen consumption (Fig. 7, *C* and *D*). Overexpression also increased extracellular acidification rate (a readout of glycolysis) but to a lesser extent, and it resulted in a larger change in oxygen consumption from baseline to stressed conditions, indicating a greater ability to meet metabolic demand (supplemental Figs. S8*D* and S7*E*). These data show that FGF1 promotes mitochondrial respiration in hepatocytes.

FGF1 exerts its effects through the FGF receptor (FGFR) tyrosine kinases. Treatment of hepatocytes with recombinant FGF1 protein is shown to reduce palmitate-induced lipid droplet formation in hepatocytes in an *Fgfr4*-dependent manner (81), yet other *Fgfr* isoforms are also expressed in liver (83). Therefore, we assessed the expression of each *Fgfr* in WT liver and observed that *Fgfr2*, *Fgfr3*, and *Fgfr4* mRNAs were expressed similarly, whereas *Fgfr1* expression was extremely low (Fig. 7*E*). *Fgfr2* and *Fgfr3* exhibited small amplitude circadian variation, peaking near the end of the light phase, and *Fgfr4* was stable over the daily cycle. To determine if the observed effect of FGF1 on mitochondrial respiration depends on activation of its receptor, we treated cells with the pan-FGFR inhibitor AZD4547 (84). At doses higher than ∼150 nM, AZD4547 inhibits all *Fgfr* isoforms (84). AZD4547 treatment at either 100 nM or 1 μM was sufficient to block the increase in oxygen consumption rate and extracellular acidification rate induced by *Fgf1* overexpression (Fig. 7*F*). Together, these data suggest that FGF1 promotes mitochondrial respiration in hepatocytes through an autocrine signaling mechanism. We surmise that the ability of FGF1 signaling to promote mitochondrial respiration depends on BMAL1 and contributes to the beneficial metabolic effects of FGF1 on liver metabolism (81, 82).

Because of the high affinity of FGF1 for heparan sulfate proteoglycans, which are components of the extracellular matrix that facilitate binding to FGFRs, it is theorized that most FGF1 does not enter the circulation but rather signals in an autocrine fashion or a paracrine fashion (80). Only FGF15/19, FGF21, and FGF23 are recognized as *bona fide* endocrine FGFs because they can bypass this mechanism. However, FGF1 is reported to circulate in mouse and human serum (85, 86). Using a commercially available ELISA, we detected FGF1 in WT serum at 0.988 ng/ml (supplemental Fig. S8*F*). Considering that FGF1 is expressed in many organs of adult mice (supplemental Fig. S8*G*), we next tested whether it can be secreted from liver using an *ex vivo* approach in which an intact liver lobe was harvested, washed in PBS several times, and incubated for 1 h in serum-free media bubbled with carbogen. The media were then concentrated with a centrifugal filter and probed *via* Western blot. The media were positive for FGF1, suggesting FGF1 can be secreted from the liver (supplemental Fig. S8*H*). Therefore, an investigation into a potential endocrine role of liver-derived FGF1 may also be warranted.

## Conclusions

Through bulk proteomics, we found that organism-wide loss of *Bmal1* impacts a greater fraction of the liver proteome than the skeletal muscle proteome. In line with our previous findings at the transcriptomic level (12), we also found that rescue of local liver and muscle clocks leads to a greater restoration of protein abundance in liver than in muscle, suggesting that the muscle clock relies more on external signals. However, our analysis in *Bmal1* KO and local clock-rescued mice under TRF showed no appreciable changes in muscle, suggesting that inputs other than feeding–fasting may be important to support muscle protein abundance during the dark and active phase. Additional circadian cues for muscle have been described; parabiosis and denervation studies suggest that neuronal innervation plays a larger role in muscle entrainment as compared with humoral factors (87, 88), and several studies demonstrate that contractile activity of the muscle, mainly through exercise, is a prominent timing cue for the muscle clock (89, 90). Thus, daily rhythms of physical activity, perhaps tied to neuronal stimulation, may substantially shape the muscle proteome. Future studies can be aimed at this open question.

Whilst we provide active phase information, many proteins have been reported to exhibit circadian variation (reviewed in Ref. (91)); therefore, we expect additional layers of complexity to be revealed once this is taken into account. We acknowledge that the bulk nature of the performed proteomics also limits sensitivity for detecting low or transiently expressed proteins. Therefore, approaches using cell type–specific labeling and enrichment (61, 92), or size-dependent fractionation, may lead to deeper proteome coverage. Yet, we successfully revealed a role for local circadian clocks in liver and muscle in supporting the expression of secreted proteins. As part of these analyses, we identified a function of liver FGF1 in supporting mitochondrial respiration in hepatocytes *via* autocrine signaling. Future studies elucidating the local and distal targets of clock-dependent secreted proteins hold promise for identifying therapeutic targets for diverse metabolic diseases associated with dysfunction of liver and skeletal muscle, such as sarcopenia, nonalcoholic fatty liver disease, and hepatocellular carcinoma (1, 93, 94, 95).

## Data Availability

The proteomics data generated in this study are deposited to the ProteomeXchange Consortium (http://proteomecentral.proteomexchange.org/cgi/GetDataset) *via* PRIDE (96) (PXD040362).

## Supplemental data

This article contains supplemental data.

## Conflict of interest

S. A. B. is a cofounder and scientific advisor of ONA Therapeutics. The authors declare no competing interests.
