# Supplementary figures and images for: Impact of Bmal1 Rescue and Time-Restricted Feeding on Liver and Muscle Proteomes During the Active Phase in Mice
Source: Mol Cell Proteomics. 2023 Oct 2;22(11):100655. doi: 10.1016/j.mcpro.2023.100655 (PMC10651687; doi:10.1016/j.mcpro.2023.100655)

**A**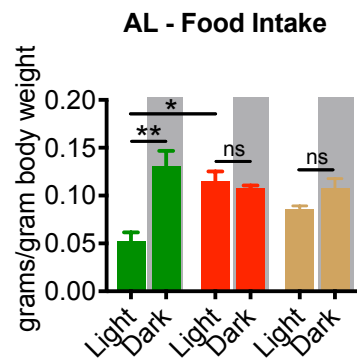**TRF - Food Intake**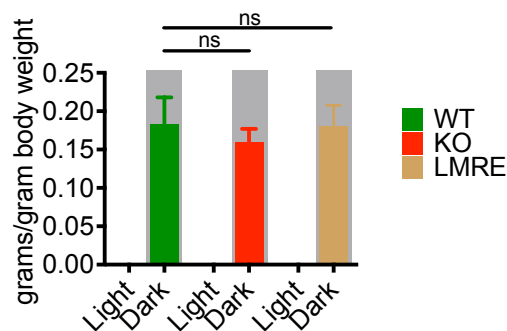**B**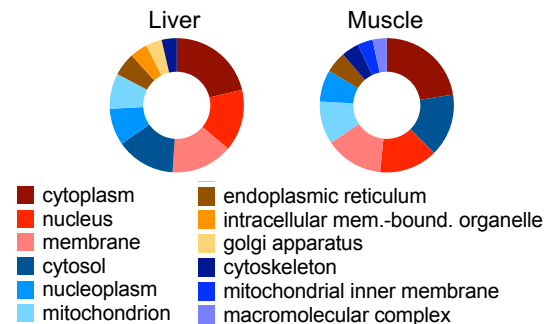**C***Bmal1*-dependent proteins in liver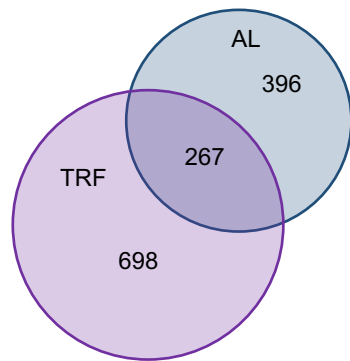**D***Bmal1*-dependent proteins in muscle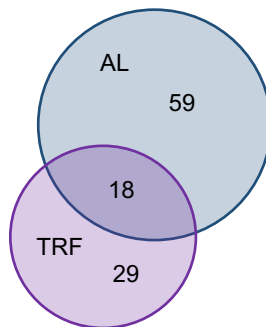

Supplement: Figure S1 [file mmc4.pdf]

**A** Non-Rescued in LMRE liver

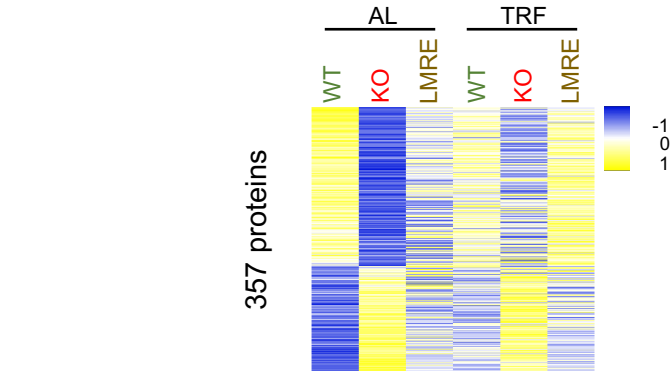

**B**

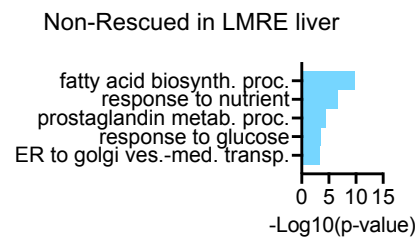

**C**

● WT ▲ KO ■ LMRE

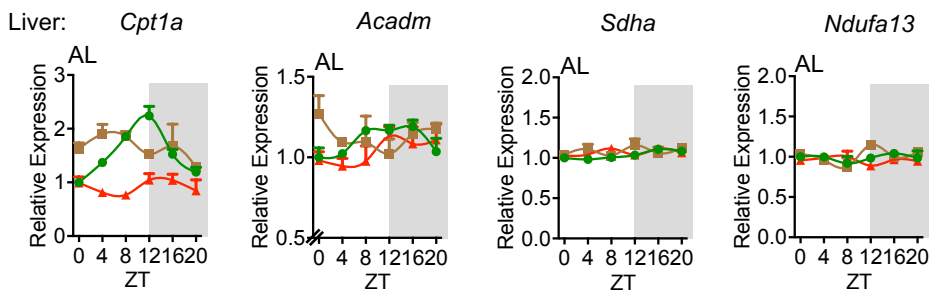

**D**

● WT ▲ KO ■ LMRE

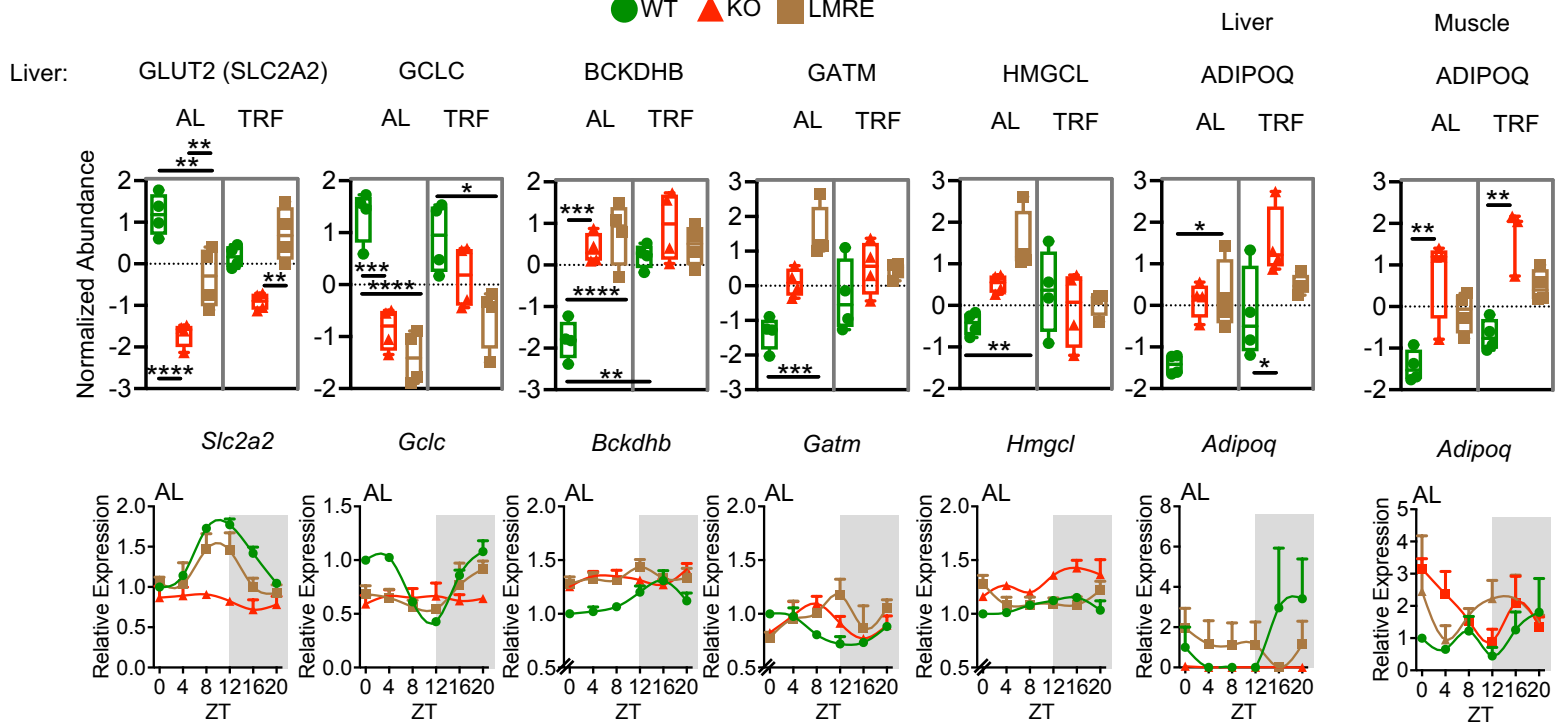

**E**

Liver: GST A, T, and M Family Proteins

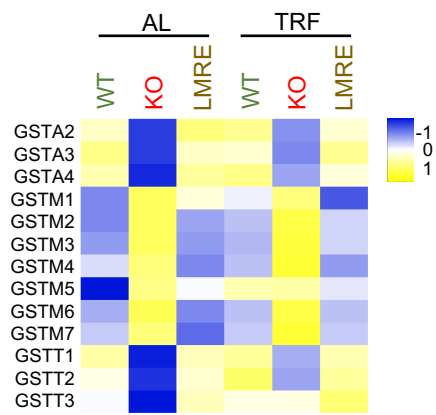

Supplement: Figure S3 [file mmc6.pdf]

**A**

● WT ▲ KO ■ LMRE

Non-Rescued: *de novo* lipogenesis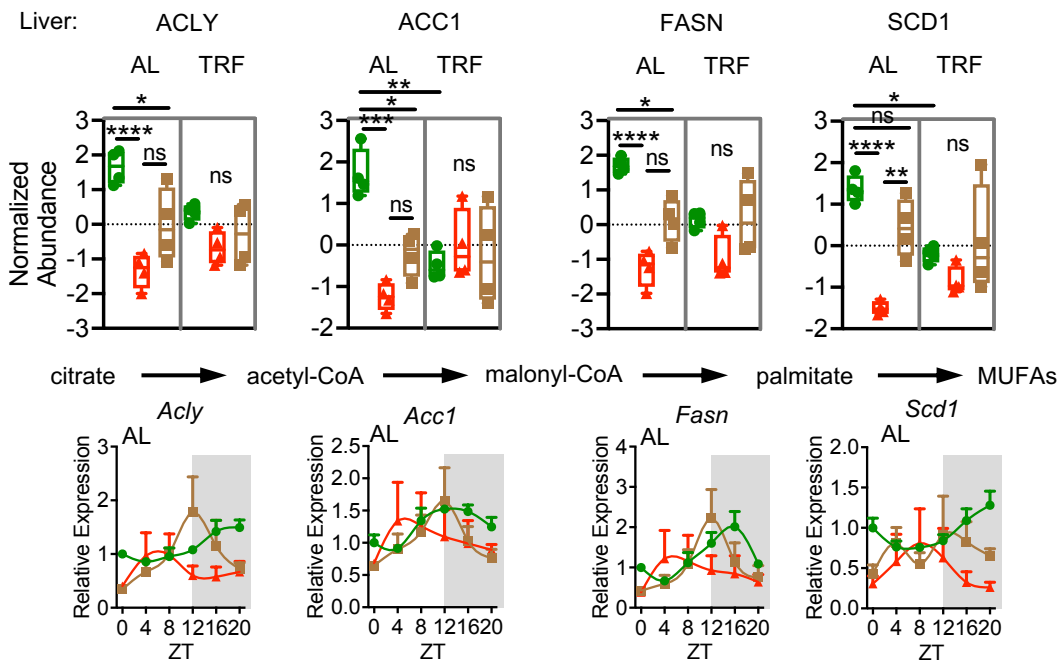**B**

● WT ▲ KO ■ LMRE

Liver: SREBF1 (SREBP)

*Srebf1*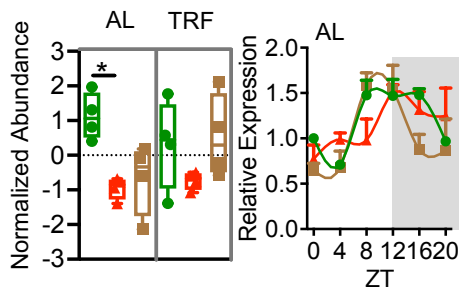

Supplement: Figure S4 [file mmc7.pdf]

# A

## Non-Rescued in LMRE muscle

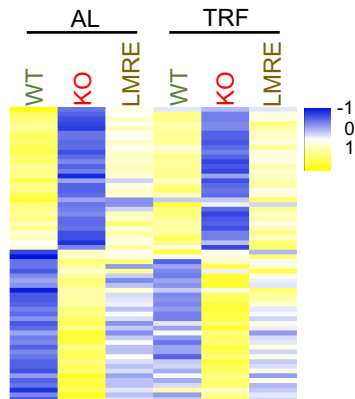

# B

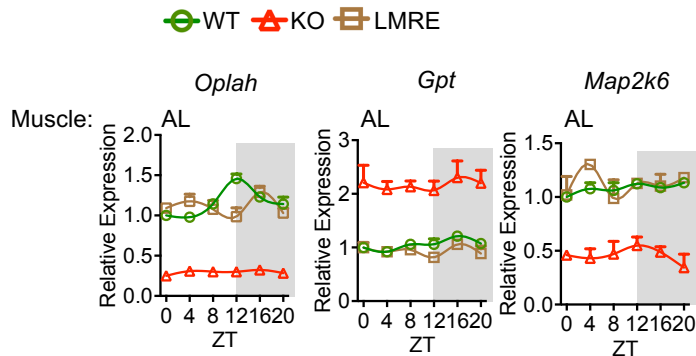

# C

## Non-Rescued in LMRE muscle

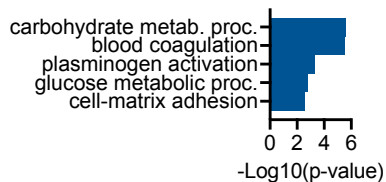

Supplement: Figure S5 [file mmc8.pdf]

**A**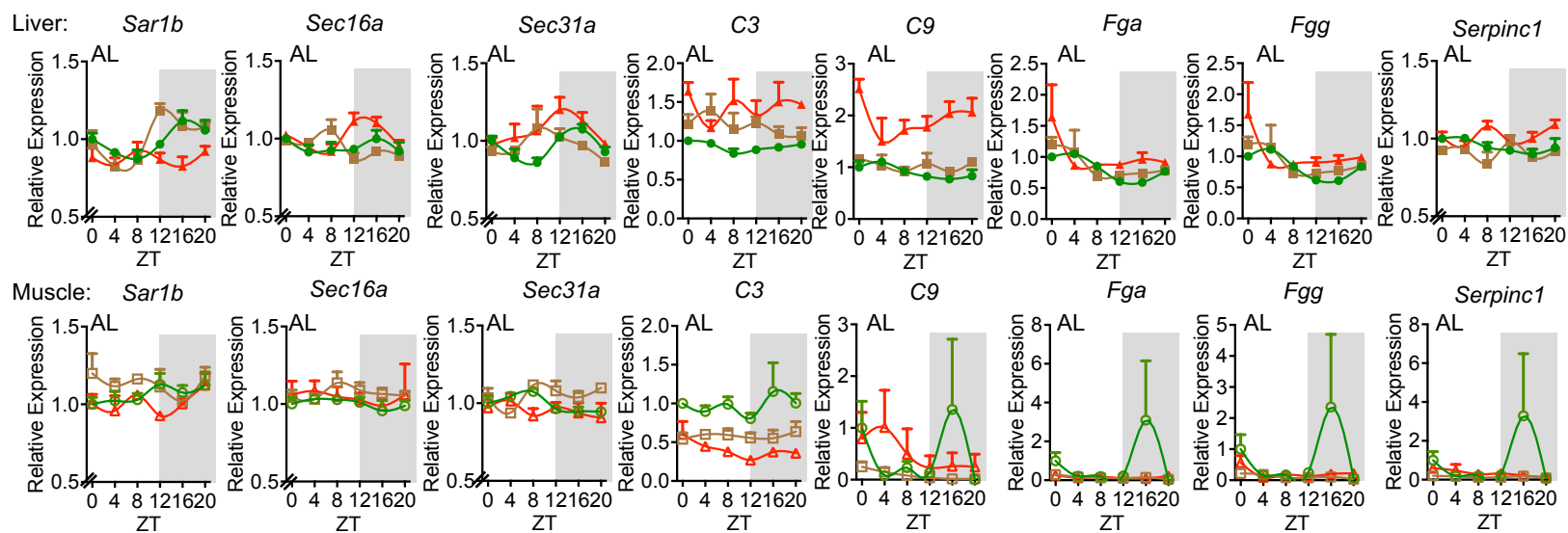**B**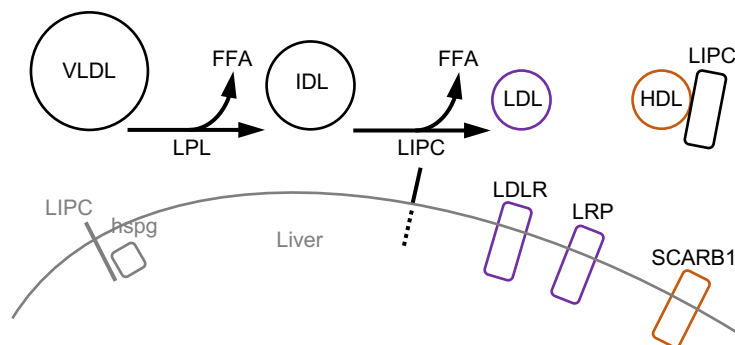**C**

● WT ▲ KO ■ LMRE

**Liver: Triglyceride Hydrolyzing Enzymes**

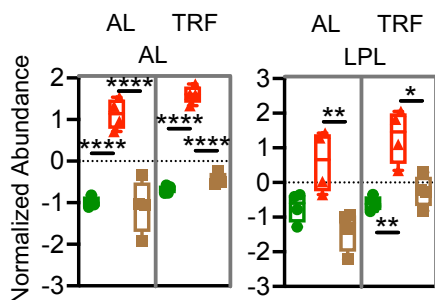

**Lipoprotein Receptors**

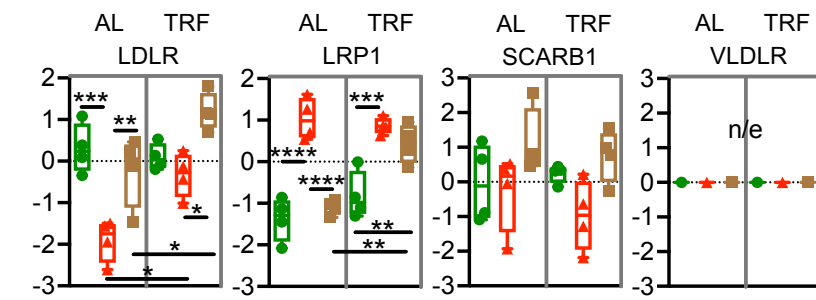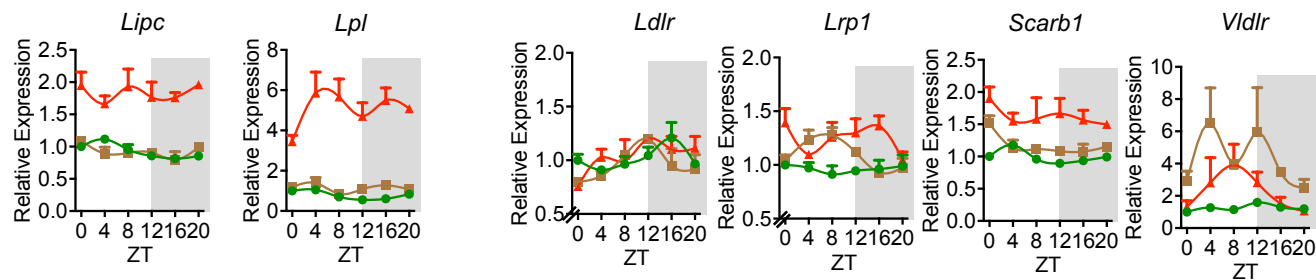

Supplement: Figure S7 [file mmc10.pdf]

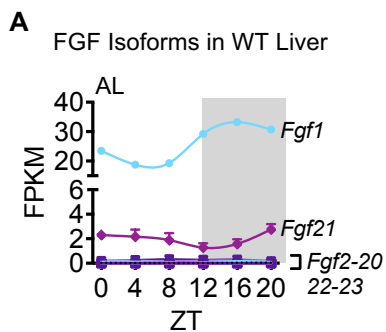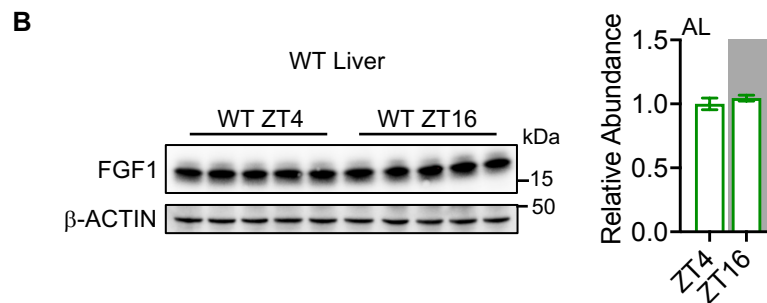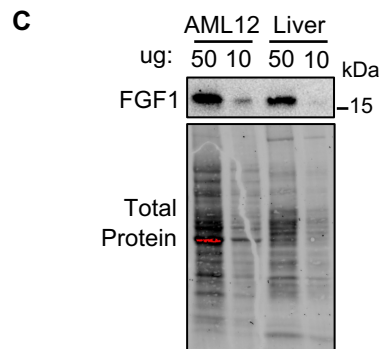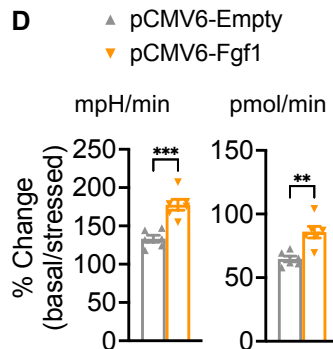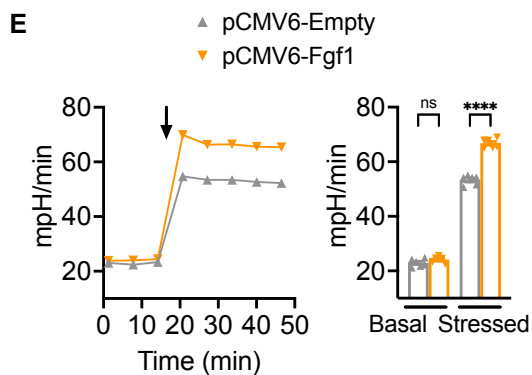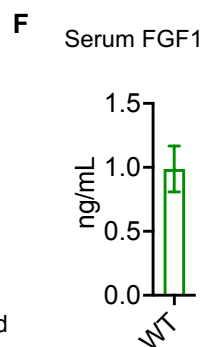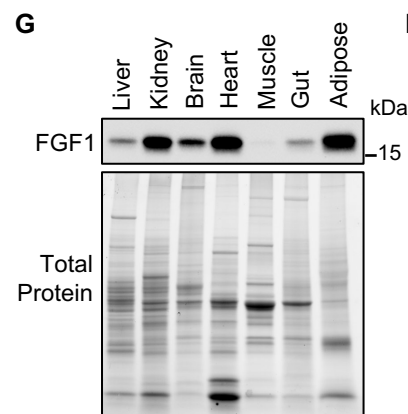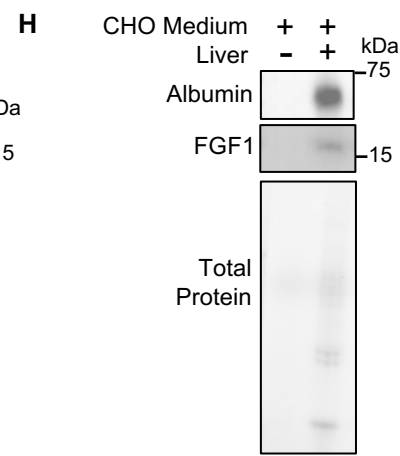

Supplement: Figure S8 [file mmc11.pdf]
