# Supplementary material for: Impact of Bmal1 Rescue and Time-Restricted Feeding on Liver and Muscle Proteomes During the Active Phase in Mice
Source: Mol Cell Proteomics. 2023 Oct 2;22(11):100655. doi: 10.1016/j.mcpro.2023.100655 (PMC10651687; doi:10.1016/j.mcpro.2023.100655)
Supplement: Figure S2 [file mmc5.pdf]

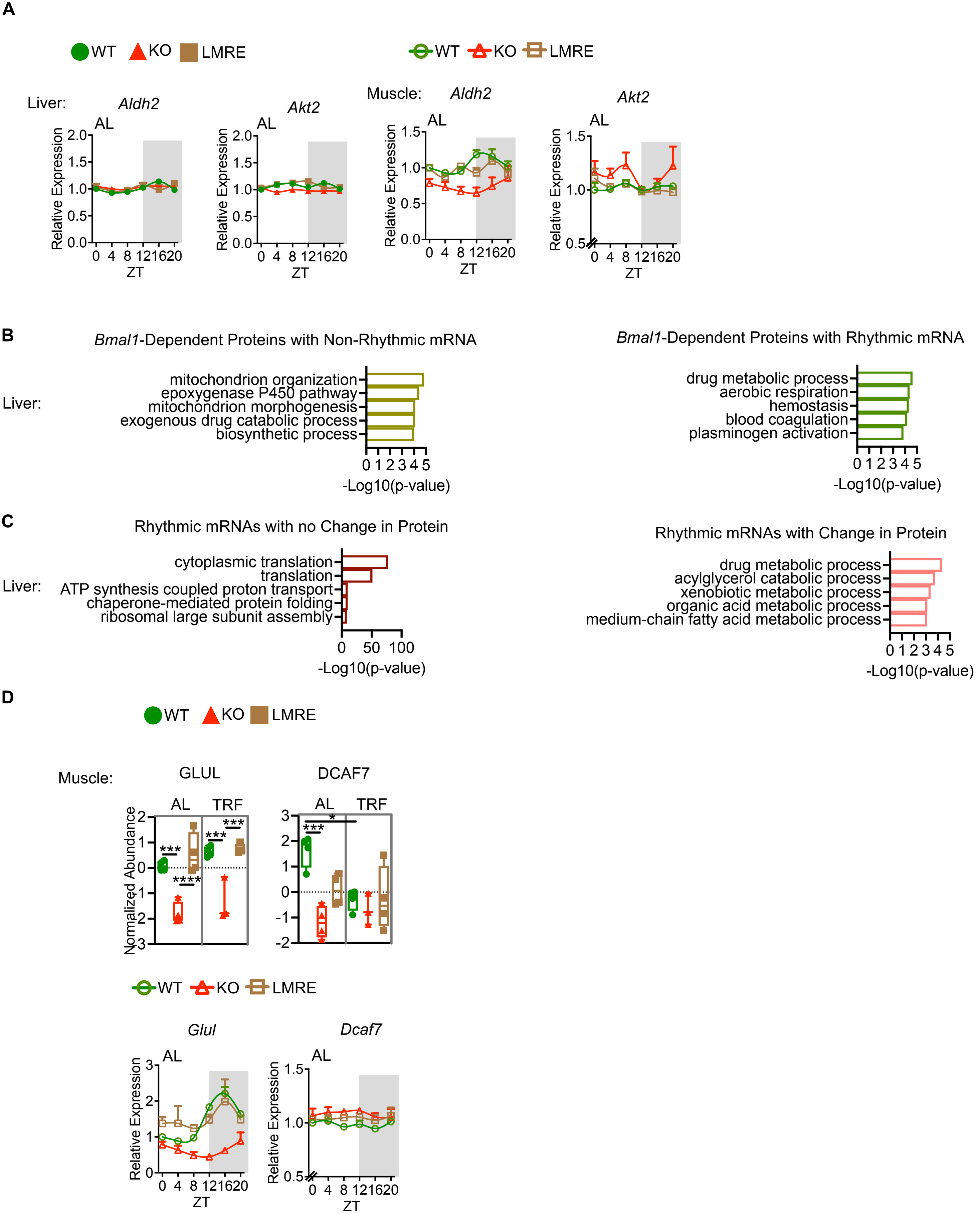
A
WT KO LMRE
WT KO LMRE
Liver: Aldh2
AL
Relative Expression
ZT
Akt2
Muscle: Aldh2
Akt2
0 4 8 121620
B
Bmal1-Dependent Proteins with Non-Rhythmic mRNA
Liver:
mitochondrion organization
epoxygenase P450 pathway
mitochondrion morphogenesis
exogenous drug catabolic process
biosynthetic process
-Log10(p-value)
Bmal1-Dependent Proteins with Rhythmic mRNA
drug metabolic process
aerobic respiration
hemostasis
blood coagulation
plasminogen activation
-Log10(p-value)
C
Rhythmic mRNAs with no Change in Protein
Liver:
cytoplasmic translation
translation
ATP synthesis coupled proton transport
chaperone-mediated protein folding
ribosomal large subunit assembly
-Log10(p-value)
Rhythmic mRNAs with Change in Protein
drug metabolic process
acylglycerol catabolic process
xenobiotic metabolic process
organic acid metabolic process
medium-chain fatty acid metabolic process
-Log10(p-value)
D
WT KO LMRE
Muscle: GLUL
AL TRF
Normalized Abundance
DCAF7
WT KO LMRE
Glul
Dcaf7
AL
Relative Expression
ZT
