# Supplementary material for: Impact of Bmal1 Rescue and Time-Restricted Feeding on Liver and Muscle Proteomes During the Active Phase in Mice
Source: Mol Cell Proteomics. 2023 Oct 2;22(11):100655. doi: 10.1016/j.mcpro.2023.100655 (PMC10651687; doi:10.1016/j.mcpro.2023.100655)

A

Ribosomal Proteins

Bmal1 Dependent in ad libitum	Bmal1 Dependent in TRF	Regulated AL vs TRF in LMRE
40S:	40S:	40S:
	RPS12	RPS10
	RPS13	RPS12
	RPS23	RPS25
	RPS27L	RPS26
	RPS28	RPS27L
	RPS3	RPS6
	RPS3A	
	RPS5	
60S:	60S:	60S:
RLP13	RPLP0	RPL10A
	RPLP1	RPL11
	RPLP2	RPL12
	RPL27A	RPL17
	RPL28	RPL19
	RPL31	RPL23
	RPL35A	RPL35A
	RPL36	RPL36A
	RPL37	RPL37
	RPL8	
	RPL9	
28S:	28S:	28S:
	MRPS10	MRPS10
	MRPS17	MRPS12
	MRPS22	MRPS21
	MRPS26	MRPS22
	DAP3	MRPS35
	MRPS30	
	MRPS31	
	MRPS33	
	MRPS5	
	MRPS6	
39S:	39S:	39S:
	MRPL1	MRPL12
	MRPL12	MRPL21
	MRPL19	MRPL27
	MRPL20	MRPL30
	MRPL23	MRPL4
	MRPL27	MRPL48
	MRPL30	MRPL50
	MRPL32	MRPL53
	MRPL37	
	MRPL39	
	MRPL40	
	MRPL42	
	MRPL46	
	MRPL54	

B

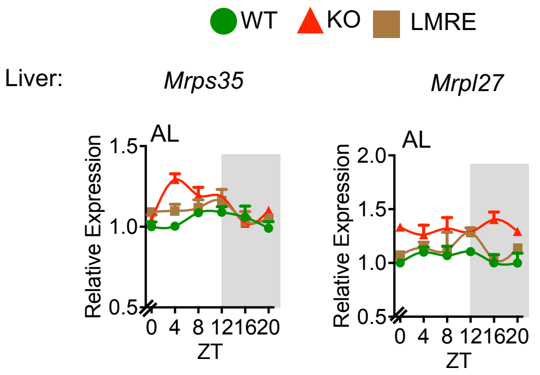

Supplement: Figure S6 [file mmc9.pdf]
